# Chemical profiling of volatile compounds of the essential oil of grey-leaved rockrose (*Cistus albidus* L.) and its antioxidant, anti-inflammatory, antibacterial, antifungal, and anticancer activity *in vitro* and *in silico*

**DOI:** 10.3389/fchem.2024.1334028

**Published:** 2024-02-15

**Authors:** Amine Elbouzidi, Mohamed Taibi, Salah Laaraj, El Hassania Loukili, Mounir Haddou, Naoufal El Hachlafi, Hanae Naceiri Mrabti, Abdellah Baraich, Reda Bellaouchi, Abdeslam Asehraou, Mohammed Bourhia, Hiba-Allah Nafidi, Yousef A. Bin Jardan, Khalid Chaabane, Mohamed Addi

**Affiliations:** ^1^ Laboratoire d’Amélioration des Productions Agricoles, Biotechnologie et Environnement (LAPABE), Faculté des Sciences, Université Mohammed Premier, Oujda, Morocco; ^2^ Euro-Mediterranean University of Fes (UEMF), Fes, Morocco; ^3^ Centre de l’Oriental des Sciences et Technologies de l’Eau et de l’Environnement (COSTEE), Université Mohammed Premier, Oujda, Morocco; ^4^ Regional Center of Agricultural Research of Tadla, National Institute of Agricultural Research (INRA), Rabat, Morocco; ^5^ Laboratory of Microbial Biotechnology and Bioactive Molecules, Faculty of Sciences and Technologies Faculty, Sidi Mohamed Ben Abdellah University, Fes, Morocco; ^6^ High Institute of Nursing Professions and Health Techniques, Casablanca, Morocco; ^7^ Laboratory of Bioresources, Biotechnology, Ethnopharmacology and Health, Faculty of Sciences, Mohammed First University, Oujda, Morocco; ^8^ Laboratory of Biotechnology and Natural Resources Valorization, Faculty of Sciences of Agadir, Ibnou Zohr University, Agadir, Morocco; ^9^ Department of Food Science, Faculty of Agricultural and Food Sciences, Laval University, Quebec City, QC, Canada; ^10^ Department of Pharmaceutics, College of Pharmacy, King Saud University, Riyadh, Saudi Arabia

**Keywords:** *Cistus albidus* L., essential oil, antioxidant, antimicrobial, anti-inflammatory, anticancer, breast cancer

## Abstract

*Cistus albidus*: L., also known as Grey-leaved rockrose and locally addressed as šṭab or tûzzâla lbîḍa, is a plant species with a well-established reputation for its health-promoting properties and traditional use for the treatment of various diseases. This research delves into exploring the essential oil extracted from the aerial components of *Cistus albidus* (referred to as CAEO), aiming to comprehend its properties concerning antioxidation, anti-inflammation, antimicrobial efficacy, and cytotoxicity. Firstly, a comprehensive analysis of CAEO’s chemical composition was performed through Gas Chromatography-Mass Spectrometry (GC-MS). Subsequently, four complementary assays were conducted to assess its antioxidant potential, including DPPH scavenging, β-carotene bleaching, ABTS scavenging, and total antioxidant capacity assays. The investigation delved into the anti-inflammatory properties via the 5-lipoxygenase assay and the antimicrobial effects of CAEO against various bacterial and fungal strains. Additionally, the research investigated the cytotoxic effects of CAEO on two human breast cancer subtypes, namely, MCF-7 and MDA-MB-231. Chemical analysis revealed camphene as the major compound, comprising 39.21% of the composition, followed by α-pinene (19.01%), bornyl acetate (18.32%), tricyclene (6.86%), and melonal (5.44%). Notably, CAEO exhibited robust antioxidant activity, as demonstrated by the low IC_50_ values in DPPH (153.92 ± 4.30 μg/mL) and β-carotene (95.25 ± 3.75 μg/mL) assays, indicating its ability to counteract oxidative damage. The ABTS assay and the total antioxidant capacity assay also confirmed the potent antioxidant potential with IC_50_ values of 120.51 ± 3.33 TE μmol/mL and 458.25 ± 3.67 µg AAE/mg, respectively. In terms of anti-inflammatory activity, CAEO displayed a substantial lipoxygenase inhibition at 0.5 mg/mL. Its antimicrobial properties were broad-spectrum, although some resistance was observed in the case of *Escherichia coli* and *Staphylococcus aureus*. CAEO exhibited significant dose-dependent inhibitory effects on tumor cell lines *in vitro*. Additionally, computational analyses were carried out to appraise the physicochemical characteristics, drug-likeness, and pharmacokinetic properties of CAEO’s constituent molecules, while the toxicity was assessed using the Protox II web server.

## 1 Introduction

Carcinogenesis is a multifaceted process that encompasses a range of risk factors, including genetic predisposition and environmental causes. Annually, there is a significant increase in the number of fatalities caused by cancer, positioning cancer as one of the primary global causes of mortality. Although many cancers do not necessarily lead to death, they considerably impair patients’ quality of life and entail high treatment costs (Łukasiewicz et al., 2021). Breast cancer is a prevalent form of cancer that affects women on a global scale. Breast cancer is a condition characterized by the growth of malignant cells in the breast tissue. While breast cancer predominantly affects women, it can also occur in men, but with significantly lower incidence (Kumar et al., 2015). Approximately 1 in 12 women may develop breast cancer during their lives. Among women, breast cancer is the primary cause of death associated with cancer. Some 685,000 women died of breast cancer in 2020. These findings show the substantial impact of breast cancer, underlining the critical need for viable preventive and treatment strategies. Numerous risk factors, most notably age, have been linked to breast cancer, and the disease’s prevalence is growing. In addition, a family history of breast cancer is a significant risk factor, suggesting a possible genetic susceptibility to the disease (Sun et al., 2017; Alfian et al., 2022). Lifestyle choices also play a crucial role, as alcohol consumption and obesity increase the risk of developing a breast tumor (Islami et al., 2018). Hormonal exposure, especially estrogen, may impact the chance of developing breast cancer. It is essential to recognize that various environmental factors, including a wide range of external influences, can also contribute to the proliferation of breast tumors (Strumylaitė et al., 2010). The approach to breast cancer treatment is determined by size, stage, and specific type of cancer. Treatment options include surgery, radiotherapy, chemotherapy, hormone therapy and targeted therapies. These modalities are designed to address specific aspects of the disease, including oxidative stress, as highlighted by (Zugazagoitia et al., 2016; Garcia-Oliveira et al., 2021).

Oxidative stress is a biological occurrence characterized by the buildup of reactive oxygen species (ROS), which are sometimes referred to as free radicals, inside the body. As a result, cellular structures are harmed (Sailaja Rao et al., 2011). This process takes place when these unstable free radicals accumulate and then induce damage to DNA, proteins, and lipids present in cells (Sailaja Rao et al., 2011). Extensive scientific research has shown a correlation between oxidative stress and breast cancer (Elbouzidi et al., 2022; Elbouzidi et al., 2023; Taibi et al., 2023b). Oxidative stress is believed to significantly impact the growth and advancement of breast cancers through many pathways. An example of such a mechanism entails the occurrence of DNA damage in breast cells, which can be ascribed to the detrimental effects of oxidative stress (Elbouzidi et al., 2022).

The body’s inflammatory response acts as a defensive mechanism against hazardous stimuli, including allergens or tissue injuries. However, an uncontrolled inflammatory reaction is a primary cause of a broad spectrum of disorders, including allergies, cardiovascular dysfunctions, metabolic syndrome, cancer, and autoimmune diseases (Boshtam et al., 2017; Dobros et al., 2022). This not only places a significant economic burden on individuals but also impacts society at large. Various medications, such as steroids, nonsteroidal anti-inflammatory drugs, and immunosuppressants, are employed to control and suppress inflammatory crises. Unfortunately, these medications come with associated adverse effects (Bouyahya et al., 2022). In practice, the aim is to administer the minimum effective dose that provides the highest efficacy while minimizing undesired side effects (Mack, 2018). Consequently, incorporating natural anti-inflammatory agents from medicinal plants into medication therapy becomes essential to enhance pharmacological response and minimize adverse effects (Singh et al., 2019).

The advent of antibiotics in the early 19th century marked a transformative period in medicine, significantly extending human life expectancy (Achinas et al., 2019). However, current scientific findings indicate a shift towards a post-antibiotic era, raising concerns about the potential return to a time before antibiotics, leading to widespread outbreaks of serious epidemic diseases, particularly those caused by bacterial infections (Abdallah, 2016; El Hachlafi et al., 2023a). Medicinal plants have become increasingly recognized as valuable repositories of therapeutic compounds capable of addressing various illnesses, especially human infections caused by pathogenic microorganisms, including multidrug-resistant pathogens (MDR) (Boren et al., 2020). In literature, a growing body of research highlights the antimicrobial effectiveness of natural substances against microorganisms. Compounds derived from medicinal plants, such as essential oils are now acknowledged as significant reservoirs for novel antimicrobial agents targeting MDR pathogens (Boren et al., 2020).

*Cistus albidus* is a species of flowering plant in the Cistaceae family (Guzmán and Vargas, 2005). It is commonly known as white-leaved rockrose or whitish rockrose. *Cistus albidus* represents one of the approximately 20 species within the *Cistus* genus (Guzmán and Vargas, 2005). It is widely believed that this name references the distinctive woody capsule fruits. Native to the Mediterranean region and typically ranging in height from 50 to 250 cm (Schmidt, 1999), this shrub derives its name “*albidus*” not from the color of its blossoms, but due to the presence of fine white hairs (trichomes) that densely cover its leaves (Raus de Baviera et al., 2023). *Cistus albidus* can be recognized by its morphological features, which include five sepals, petals that vary in color from pink to purple, a large number of up to 150 yellow stamens, pollen with a thin exine measuring up to 1.4 µm in thickness, an elongated style that is equal to or longer than the stamens, and tricellular polysperm capsules. The flowers are hermaphroditic, actinomorphic, and hypogynous. They usually bloom from February to July and have a diameter of four to 6 cm. They can appear individually or in clusters, with an average count of five to seven (Guzmán and Vargas, 2009). Ethnopharmacological surveys conducted in different countries showed numerous traditional applications of *C. albidus*. Different plant parts of *C. albidus* were used against diabetes and various inflammatory diseases, respiratory disorders such as bronchitis, pneumonia, and respiratory infections, wounds, urinary complications, gastric and intestinal ulcers (Tahiri et al., 2017; Tomou et al., 2022).

The plant has enjoyable biological activities, including antioxidant, anti-inflammatory, and antimicrobial activity (Haouat et al., 2013; Bouyahya et al., 2016; Fadel et al., 2020). These effects are mainly attributed to its chemical composition rich in secondary metabolites.

There is an increasing interest in exploring essential oils as an alternate approach to mitigate the adverse effects linked to traditional therapies. The main aim of our work is to conduct a phytochemical examination of the essential oil derived from *C. albidus* (CAEO) by utilizing gas chromatography combined with mass spectrometry (GC/MS). In addition, we are conducting research on the antioxidant, antibacterial, and antifungal characteristics of CAEO. Furthermore, we are evaluating its potential as an anti-inflammatory agent using a 5-Lipoxygenase assay. In addition, we are investigating the cytotoxic properties of this essential oil on specific breast cancer cell lines, specifically MCF-7 and MDA-MB-231. To gain insights into the mechanisms of action of the bioactive compounds in CAEO, we conducted *in silico* receptor-ligand docking studies to predict the various biological activities under investigation. Additionally, we explored the pharmacokinetic properties of CAEO’s components and examined potential toxicological endpoints. The goal of this thorough evaluation is to close information gaps about the possible advantages of CAEO in treating breast cancer. As an antioxidant, antibacterial, antifungal, and anti-inflammatory agent. The outcomes of this study may open promising avenues for future research and therapeutic applications.

## 2 Results and discussion

### 2.1 Chemical profile of *C. albidus* essential oil (CAEO)

**FIGURE 1:**
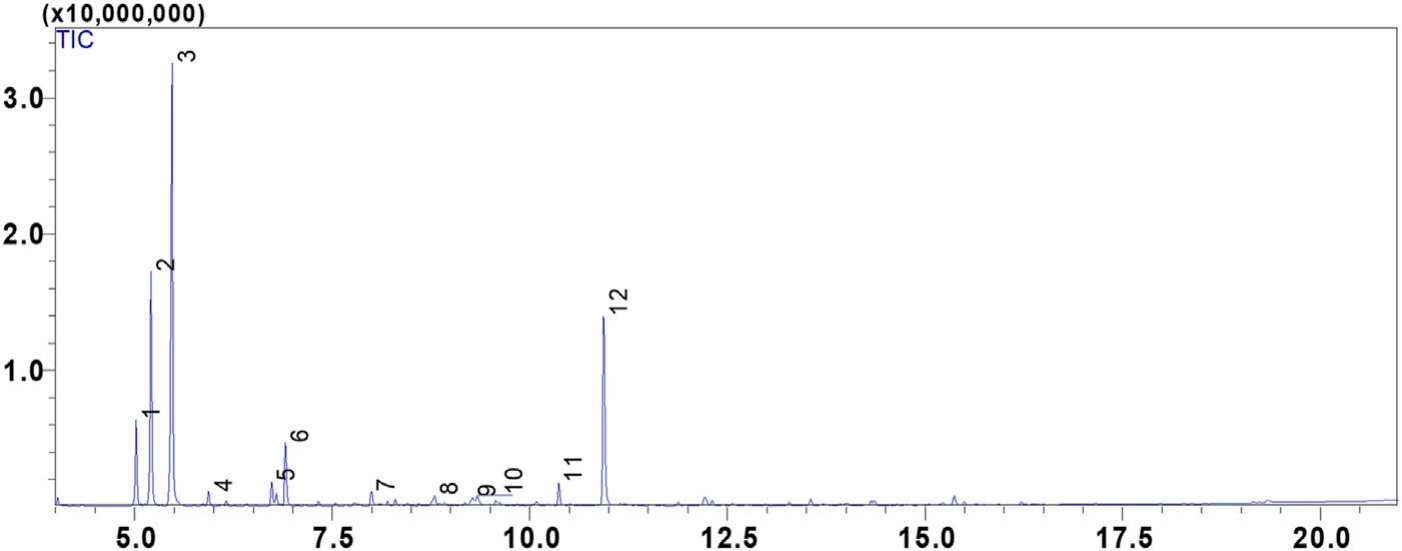
GC/MS chromatogram of the compounds identified in the essential oil of *Cistus albidus* (CAEO).

In our study, we utilized hydrodistillation to extract essential oils from the fresh aerial parts of *C. albidus*. This method yielded significantly higher extraction rates, ranging from 0.5% to 0.8% (*w/w*). In contrast, prior research employing steam distillation yielded comparatively low to modest yields, typically falling within the range of 0.01%–0.1% (*w/w*) (Llusià et al., 2010; Bechlaghem et al., 2019). The qualitative analysis of *C. albidus* essential oil (CAEO) reveals that camphene is the predominant compound, comprising approximately 39.21% of the composition. The primary constituents subsequent to camphene are α-Pinene (19.01%), bornyl acetate (18.32%), tricyclene (6.86%), and melonal (5.44%) (Figure 1; Table 1). Additionally, minor quantities of other compounds were identified, including β-pinene, *m*-cymene, linalyl alcohol, camphor, borneol, cis-β-Terpineol, and linalool acetate (Table 1). Notably, our findings indicate significant differences in the concentrations of these major components when compared to previously published data on the composition of Cistus essential oils. An earlier study conducted by Palá-Paúl et al. examined the essential oil extracted from *C. albidus* leaves collected in Spain during the winter season. The study identified the presence of zingiberene (14.8%), aromadendrene (10.6%), ar-curcumene (10.6%), and guaiol (6.5%) in the oil (Palá-Paúl et al., 2005). Likewise, the essential oil derived from spring leaves had the same primary components as the essential oil from winter leaves, but there were minor differences in quantity. The compounds identified in the study were aromadendrene (9.2%), ar-curcumene (8.8%), zingiberene (8.0%), and guaiol (7.0%) (Palá-Paúl et al., 2005). The composition of *C. albidus* flower oil closely paralleled that of the leaf oils, with just small differences (Palá-Paúl et al., 2005). The main components of the floral oil were α-cadinol (8.4%), zingiberene (8.1%), curcuphenol (6.0%), δ-cadinene (5.5%), 10-epi-γ-eudesmol (5.4%), and β-caryophyllene (5.3%) (Palá-Paúl et al., 2005). The obtained results in this current study are inconsistent with those reported in the study of Belghachem et *al*., the analysis of the essential oil derived from the aerial parts of *C. albidus* in Algeria revealed a unique composition consisting exclusively of sesquiterpenes. This composition comprised 15 sesquiterpene hydrocarbons, accounting for 48.6% of the total, and 16 oxygenated sesquiterpenes, which constituted 44.8% of the oil. The primary constituents identified in this essential oil included epi-αbisabolol (11.4%), β-bourbonene (8.7%), ar-curcumene (8.3%), α-zingiberene (7.4%), γ-muurolene (5.6%), 14-hydroxy-α-muurolene (5.2%), β-caryophyllene (4.5%), among others (Bechlaghem et al., 2019). Hence, (Morales-Soto et al., 2015), have shown that camphor (43.86%), E-caryophyllene (19.26%), eucalyptol (19.14%), and β-bourbonene (13.27%) were reported as the main volatile components of the aerial parts of *Cistus salviifolius* from Spain. Using solid-phase microextraction (SPME), volatile chemicals from C. criticus leaves and flowers were mostly made up of monoterpene and sesquiterpene hydrocarbons. Among these, α-pinene, camphene, and cubebene were the most abundant constituents. The oil obtained from the leaves during hydrodistillation was primarily composed of oxygenated diterpenes and diterpene hydrocarbons, with the main constituents being manoyl oxide and sclarene. The flower-derived oil was primarily composed of oxygenated diterpenes and diterpene hydrocarbons, with the main constituents being manoyl oxide and abietatriene.

**TABLE 1:** Phytochemical profile of CAEO.

N° ^a^	R_i_ ^c^	R_i_ lit ^d^	Compounds ^b^	Chemical structure	RT (min)	Peak (%)	Identification
1	923	923	Tricyclene	C_10_H_16_	5.019	6.86	MS, R_i_
2	931	933	α-Pinene	C_10_H_16_	5.207	19.01	MS, R_i_
3	942	944	Camphene	C_10_H_16_	5.472	39.21	MS, R_i_
4	959	973	β-Pinene	C_10_H_16_	5.935	1.27	MS, R_i_
5	985	984	*m*-Cymene	C_10_H_14_	6.734	2.13	MS, R_i_
6	991	992	Melonal	C_9_H_16_O	6.908	5.44	MS, R_i_
7	1021	1021	Linalyl alcohol	C_10_H_18_O	7.996	1.63	MS, R_i_
8	1041	1042	Camphor	C_10_H_16_O	8.794	1.43	MS, R_i_
9	1052	1146	Borneol	C_10_H_18_O	9.274	1.04	MS, R_i_
10	1053	1054	*cis*-β-Terpineol	C_10_H_18_O	9.335	1.08	MS, R_i_
11	1336	1336	Linalool acetate	C_12_H_20_O_2_	10.367	2.58	MS, R_i_
12	1363	1360	Bornyl acetate	C_12_H_20_O_2_	10.936	18.32	MS, R_i_
					Identified compounds		Percentage
		*Hydrocarbon monoterpenes*			5		68.48
		*Oxygenated monoterpenes*			5		10.62
		*Hydrocarbon sesquiterpenes*			-		-
		*Oxygenated sesquiterpenes*			-		-
		*Others*			2		20.9
		Total identified (%)					100

^a^ In order of elution.

^b^ Components identified by KI, and MS.

^c^ Kovats index calculated from alkanes series on the MS, capillary column (C8–C24).

^d^ Kovats index/Retention index from data libraries (NIST).

Previous investigations on *C. albidus* (Maccioni et al., 2007; Llusià et al., 2010; Bechlaghem et al., 2019) have consistently found relatively small or negligible quantities of monoterpenes in leaves, flowering tops, and flowers, whereas sesquiterpenes were found to be more abundant. Our research revealed a significant presence of hydrocarbon monoterpenes (comprising 68.48% of the composition) and oxygenated monoterpenes (constituting 10.62%). In support of our findings, a recent investigation conducted by Hachlafi et *al*. (2023) highlighted the potent pharmacological properties associated with camphene. These properties encompass antibacterial, antifungal, anticancer, antioxidant, antiparasitic, antidiabetic, anti-inflammatory, and hypolipidemic attributes (Hachlafi et al., 2023). The bicyclic terpenoid α-pinene has anti-inflammatory and anti-apoptotic properties. These findings underscore the dynamic nature of essential oil compositions in *C. albidus*, with variations observed not only between different seasons but also in comparison to existing literature.

### 2.2 Physiochemical, drug-likeness, and pharmacokinetic properties (ADME) of CAEO

**FIGURE 2:**
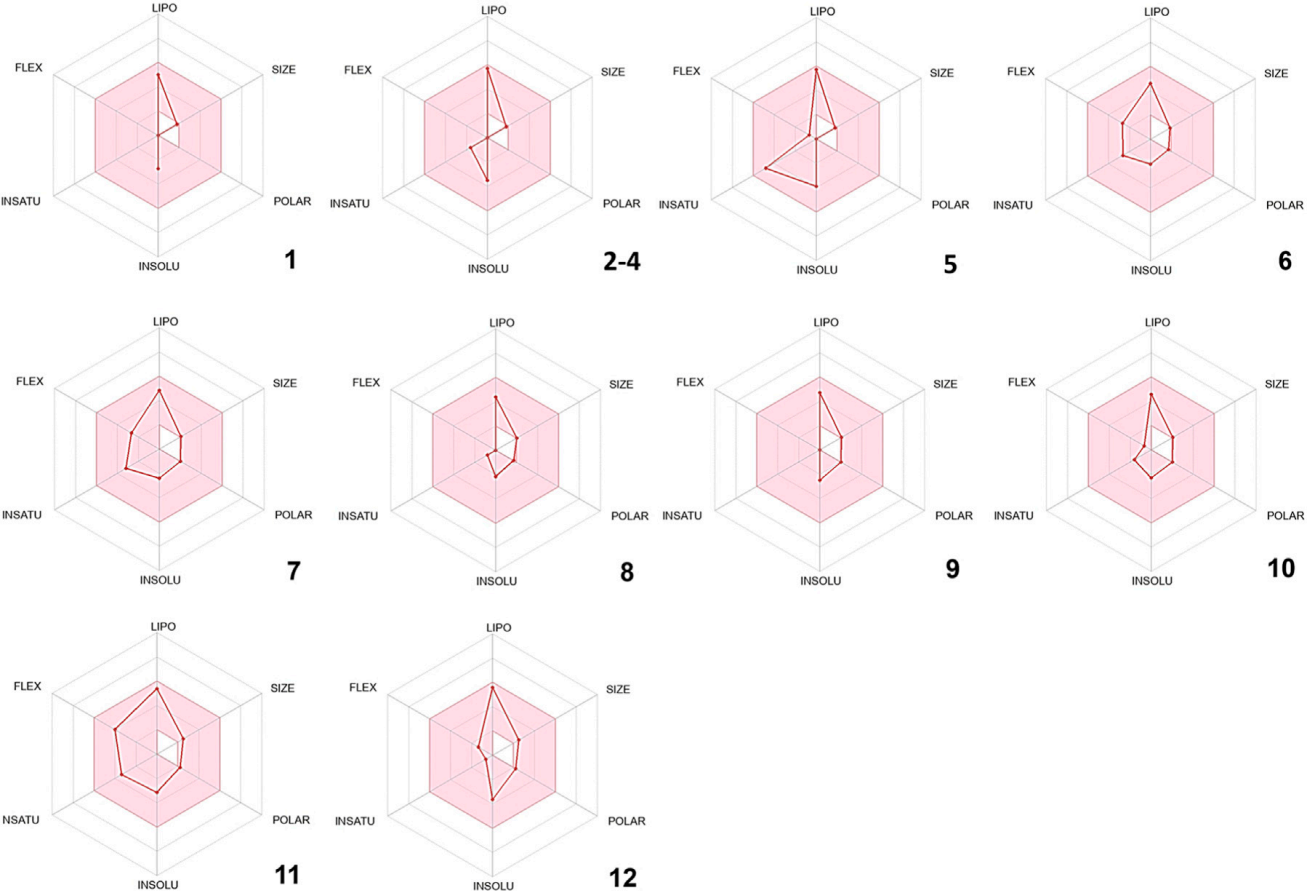
Bioavailability radars of the volatile composition of CAEO. **(1)** Tricyclene, **(2)** α-Pinene, **(3)** Camphene, **(4)** β-Pinene, **(5)** *m*-Cymene, **(6)** Melonal, **(7)** Linalyl alcohol, **(8)** Camphor, **(9)** Borneol, **(10)** *cis-* β-Terpineol, **(11)** Linalool acetate, **(12)** Bornyl acetate.

*In silico* drug-likeness assessments are pivotal for streamlining drug discovery by efficiently filtering and prioritizing potential candidates (Tian et al., 2015). These computational techniques save time and resources, minimize the experimental load, and allow for the early discovery of compounds with favorable pharmacokinetic features and target interactions (Ursu et al., 2011; Jia et al., 2020). In order to comply with Lipinski’s rule of five, certain physical and chemical attributes must be met. These include having less than 5 hydrogen bond donors, fewer than 10 hydrogen bond acceptors, no more than 10 nitrogen or oxygen atoms, a molecular weight of less than 500 Da, and an MLOGP value less than or equal to 4.15. Remarkably, all the phytoconstituents mentioned above meet Lipinski’s rule of five (Supplementary Table S2). Furthermore, other drug-likeness rules such as Veber’s and Egan’s were met by all the compounds identified in CAEO (Supplementary Table S2). As per Martin’s findings in 2005, any compound that complies with Lipinski’s rule of five is assigned a bioavailability score of 0.55 (Martin, 2005). Therefore, all the identified compounds are given a bioavailability score of 0.55. Figure 2, displays the bioavailability radars for the compounds that were identified. In these radar plots, the pink region constitutes the oral bioavailability space, and for a compound to be considered drug-like, its graph must entirely fall within this area (Figure 2). In the current study, it is noteworthy that all the phytochemicals conform to the appropriate space required for oral bioavailability, suggesting their potential suitability as drug candidates.

A medicine’s limited absorption, distribution, metabolism, and excretion, known as its ADME characteristics, can jeopardize its efficacy. Moreover, the primary challenge in drug development during clinical trials is the pharmacokinetic characteristics of a drug, which can result in substantial costs. Consequently, *in silico* methods were employed to assess ADME attributes to predict the potential suitability of *C. albidus* essential oil as a drug development candidate. In this research, various factors were taken into account, including physical and chemical properties, as well as factors related to absorption, distribution, metabolism, and excretion (Table 2). Compounds having a logS (log mol/L) value between −4 and 0 have good water solubility (Daina et al., 2017). In this context, phytochemicals are considered to have favorable water solubility, with the exception of camphene (3), β-pinene (4), and m-cymene (5), which have a moderate amount of water solubility. All phytochemical substances have high Caco-2 permeability, with log *p* values about 10–6 cm/s. As a result, they show a high percentage of absorption in the human gut, ranging from 93.16% to 96.28%. Log Kp (in cm/s) is an important measure of a compound’s capacity to penetrate the skin, especially in the context of transdermal medication administration (Chen et al., 2018). In general, a low skin permeation is confirmed when a molecule’s log Kp exceeds −2.5 cm/s (Pires et al., 2015). In our study, it was observed that seven compounds among those identified, namely, Melonal, linalyl alcohol, camphor, borneol, cis-β-terpineol, linalool acetate, and bornyl acetate, exhibited log Kp values greater than −2.5 cm/s, indicating a limited degree of skin permeation. In the context of distribution parameters, VDss, which stands for the steady-state volume of distribution, represents the theoretical volume required to uniformly disperse the entire dose of a drug or molecule in such a way that it achieves the same concentration as that found in the blood plasma (Zou et al., 2012). VDss is typically categorized as high if it surpasses 2.81 L/kg or if the log VDss exceeds 0.45 (Pires et al., 2015). Conversely, it is considered low if it falls below 0.71 L/kg or if the log VDss is less than −0.15 (Pires et al., 2015). In our case, all the compounds under investigation exhibit a high VDss, as indicated by log VDss values spanning from 0.047 (in the case of Linalool acetate) to 0.790 (for tricyclene) (Supplementary Table S3). This signifies that these compounds possess a significant distribution capacity, allowing them to disperse throughout the body in a manner that is conducive to achieving therapeutic concentrations in the blood plasma. The brain is shielded from external compounds by the blood-brain barrier (BBB), a crucial protective mechanism. The capacity of a drug or molecule to permeate the BBB is a pivotal factor to take into account. This parameter plays a significant role in minimizing potential toxicities and side effects, as well as in enhancing the efficacy of a drug or molecule whose intended pharmacological action is within the brain. In the case of the compounds under scrutiny, it is noteworthy that all of them exhibit a log BB value exceeding 0.3. This observation strongly suggests that the identified phytochemicals possess a high propensity for distribution into the brain, indicating their potential for affecting neurological targets and activities.

**TABLE 2:** *In silico* evaluation of the pharmacokinetic properties (ADME) of the identified components in CAEO. **(1)** Tricyclene, **(2)** α-Pinene, **(3)** Camphene, **(4)** β-Pinene, **(5)** *m*-Cymene, **(6)** Melonal, **(7)** Linalyl alcohol, **(8)** Camphor, **(9)** Borneol, **(10)** *cis-* β-Terpineol, **(11)** Linalool acetate, **(12)** Bornyl acetate.

Prediction	1	2	3	4	5	6	7	8	9	10	11	12
	ADME prediction											
	Physicochemical properties											
TPSA (Å^2^)	0.00 Å^2^	0.00 Å^2^	0.00 Å^2^	0.00 Å^2^	0.00 Å^2^	17.07 Å^2^	20.23 Å^2^	17.07 Å^2^	20.23 Å^2^	20.23 Å^2^	26.30 Å^2^	26.30 Å^2^
Absorption Parameters												
Water Solubility	−3.909	−3.733	−4.340	−4.191	−4.098	−2.852	−2.612	−2.878	−2.447	−2.055	−2.749	−3.015
Solubility Class	Soluble	Soluble	M.S.	M.S.	M.S.	Soluble	Soluble	Soluble	Soluble	Soluble	Soluble	Soluble
Caco-2 Permeability	1.363	1.380	1.387	1.385	1.526	1.499	1.493	1.509	1.494	1.489	1.172	2.086
Intestinal Absorption (%)	95.29	96.04	94.14	95.52	93.64	95.48	93.16	96.42	93.89	93.72	95.93	96.28
Log K_*p*_ (cm/s)	−4.83	−3.95	−4.13	−4.18	−3.92	−1.53	−1.73	−2.02	−2.19	−2.39	−1.91	−2.26
Distribution Parameters												
VDss	0.790	0.667	0.547	0.685	0.724	0.131	0.152	0.332	0.338	0.207	0.047	0.313
BBB Permeability	Log BB > 0.30 Yes	Log BB > 0.30 Yes	Log BB > 0.30 Yes	Log BB > 0.30 Yes	Log BB > 0.30 Yes	Log BB > 0.30 Yes	Log BB > 0.30 Yes	Log BB > 0.30 Yes	Log BB > 0.30 Yes	Log BB > 0.30 Yes	Log BB > 0.30 Yes	Log BB > 0.30 Yes
Metabolism Parameters												
CYP2D6, and CYP3A4 Substrate	No	No	No	No	No	No	No	No	No	No	No	No
CYP2D6, and CYP3A4 Inhibitors	No	No	No	No	No	No	No	No	No	No	No	No
Excretion Parameters												
Total Clearance	−0.073	0.043	0.049	0.030	0.249	0.449	0.446	0.109	1.035	1.252	1.631	1.029
Renal OCT2 Substrate	No	No	No	No	No	No	No	No	No	No	No	No

n. d.: not determined; TPSA: topological polar surface area; Water Solubility: log mol/L, M.S.: moderate solubility; VDss: log L/kg; Total Clearance: log (mL/min/kg).

When it comes to assessing metabolic parameters, it is crucial to consider the role of Cytochrome P450, a vital detoxification enzyme primarily found in the liver (Chen et al., 2019). Compounds that inhibit this enzyme can have a profound impact on drug metabolism and are typically discouraged due to potential adverse effects. Fortunately, in our study, none of the compounds were identified as inhibitors or substrates of CYP450 enzymes, particularly the CYP2D6 and CYP3A4 isoforms (Table 2). This finding suggests a reduced risk of interference with drug metabolism and supports the safety profile of these compounds.

**FIGURE 3:**
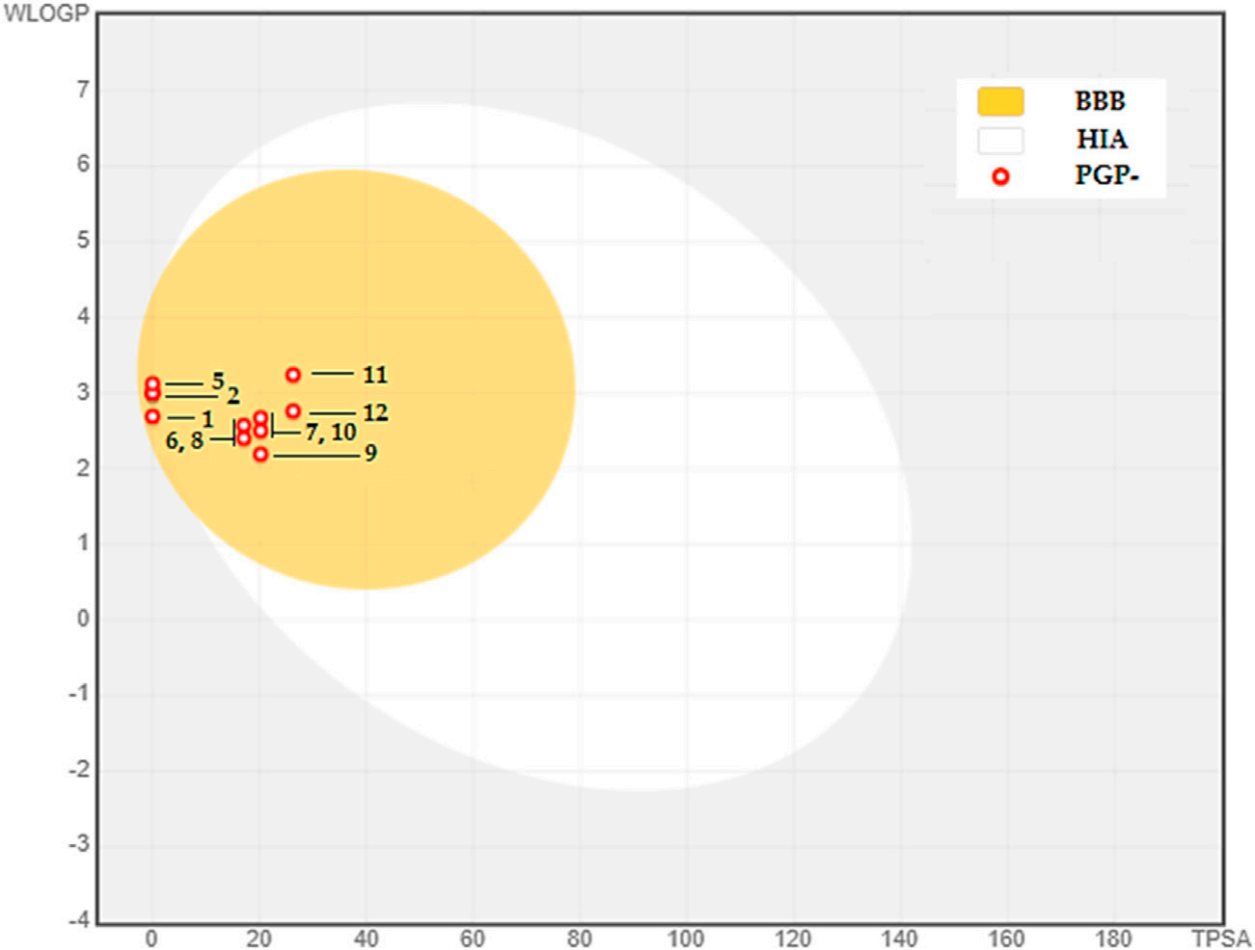
BOILED-EGG Model of the BBB permeability and GI absorption of the major compounds in CAEO. **(1)** Tricyclene, **(2)** α-Pinene, **(3)** Camphene, **(4)** β-Pinene, **(5)** *m*-Cymene, **(6)** Melonal, **(7)** Linalyl alcohol, **(8)** Camphor, **(9)** Borneol, **(10)** *cis-* β-Terpineol, **(11)** Linalool acetate, **(12)** Bornyl acetate.

The BOILED-Egg model is used as an initial tool to assess two vital factors: intestinal absorption (IA) and blood-brain barrier permeability (BBB). This evaluation mostly depends on the molecule’s lipophilicity (WLOGP) and polarity (TPSA), as stated by (Daina and Zoete, 2016). The model depicts molecules with strong capacity to be absorbed in the intestines in the white zone, whereas those with high permeability across the blood-brain barrier are located in the yolk, shown by the yellow area (see Figure 3). The graph’s dots are color-coded to indicate whether a chemical functions as a P-glycoprotein substrate. Those that are P-glycoprotein substrates are shown by blue dots, whereas those that are not are represented by red dots.

Within this particular setting, the discovered phytochemicals have demonstrated robust intestinal absorption and exceptional capacity to permeate the blood-brain barrier. Moreover, they cannot serve as substrates for P-glycoprotein, indicating their potential as very favorable candidates for diverse therapeutic uses.

### 2.3 In silico prediction of organ toxicity and toxicological-endpoints

A meticulous and comprehensive analysis was conducted to assess the toxicological properties of the primary constituents CAEO, as reported in previous studies (Banerjee et al., 2018; Elbouzidi et al., 2023; Haddou et al., 2023). The summative findings of this investigation, as presented in Supplementary Table S3, encompassed an array of toxicity endpoints, including hepatotoxicity, carcinogenicity, cytotoxicity, immunotoxicity, mutagenicity, the predictive lethal dose for 50% of the population (LD_50_), and the resulting classification according to toxicity class. The LD_50_ values serve as indicators of the acute toxicity of the compounds, where diminished values imply heightened levels of toxicity. This in-depth evaluation produced results that affirm the overall safety profile of the assessed compounds, placing them predominantly within the less toxic classifications of toxicity classes IV to VI. Notably, no substantial toxicological endpoints were observed for the majority of the compounds, signifying their relatively benign nature. However, it is pertinent to mention that an exception emerged in the case of *m*-Cymene, wherein a probability value of 0.67 indicated a potential carcinogenicity concern, thereby warranting further investigation and consideration of its application in specific contexts within the realms of toxicology and public health.

### 2.4 Molecular docking predictions of the possible mechanisms of action of CAEO compounds

In order to investigate the impact of the C. albidus essential oil (CAEO) on pharmacological activities, we used several computer-based approaches to perform molecular docking of its bioactive components with their respective molecular receptors. The strength of binding is inversely related to the numerical magnitude of binding affinity (kcal/mol). The highest docking prediction demonstrated an anticipated binding affinity, with a root mean square deviation of zero (Ouahabi et al., 2023).

In this study, we utilized a method to evaluate the binding affinities of 12 compounds present in the essential oil. The antioxidant activity of these compounds was evaluated against five proteins associated with distinct biological functions: Dihydrofolate reductase (DHFR), which exhibited antibacterial activity (PDB ID: 4M6J); Cytochrome P450 alpha-sterol demethylase (PDB ID: 1EA1), 5-Lipoxygenase (PDB ID: 1N8Q); and the anticancer activity of the epidermal growth factor receptor (EGFR), which possessed anticancer properties (Tomy et al., 2018; Khatun et al., 2). The outcomes of the molecular docking studies are displayed in a heat map table (Table 3.) utilizing a color gradient ranging from red to blue, with a transition through white (at a percentile of 50) to emphasize the energies of the docking scores. The lower energy scores, often corresponding to the score of the native ligand or a very effective inhibitor, are displayed in red, showing the most optimal matches. Blue or other hues of blue indicate higher energy values, indicating a lower affinity to the target. This approach makes it easy to identify chemical compounds with the potential to inhibit specific targets.

**TABLE 3:** The molecular free binding affinity (measured in kcal/mol) was obtained from computational simulations of the identified compounds in CAEO.

N°	Compounds	3GRS (Antioxidant)	4M6J (Antibacterial)	1EA1 (Antifungal)	1N8Q (Anti-inflammatory)	1XKK (Cytotoxicity)
			Free binding energy (Kcal/mol) *				
-	Native Ligand	−6.3	−7.0	−5.8	−6.0	−5.8
1	Tricyclene	−5.1	−5.9	−4.0	**−6.2**	−4.7
2	α-Pinene	−5.2	−5.7	−4.2	−5.6	−5.0
3	Camphene	−5.1	−5.7	−4.0	−4.7	−4.6
4	β-Pinene	−5.0	−5.7	−4.1	−4.8	−4.7
5	*m*-Cymene	−5.8	−6.3	−4.2	−5.4	−5.3
6	Melonal	−4.7	−4.9	−3.0	−4.8	−4.0
7	Linalyl alcohol	−4.9	−5.0	−3.8	−4.7	−4.5
8	Camphor	−5.3	−5.8	−4.4	−5.3	−4.8
9	Borneol	−5.2	−5.6	−4.3	−5.7	−4.6
10	*cis-* β-Terpineol	−5.4	−5.8	−4.3	−5.3	−5.1
11	Linalool acetate	−5.0	−5.2	−4.2	−5.0	−5.0
12	Bornyl acetate	−6.3	−7.0	−5.8	−6.0	−5.9

#### 2.4.1 Interactions with glutathione reductase (PDB: 3GRS)

**FIGURE 4:**
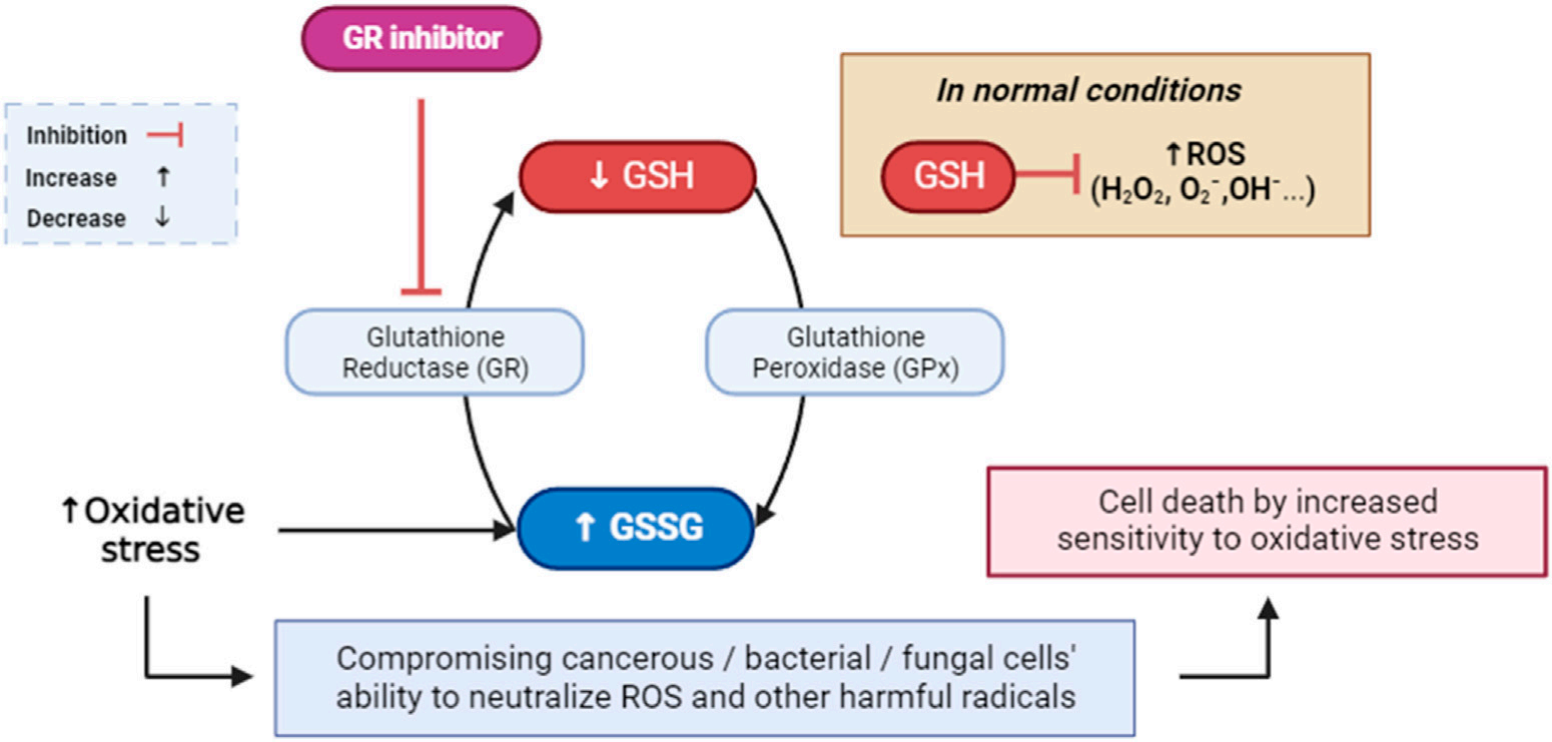
Mediated inhibition of glutathione reductase, implications for cellular defense against ROS and free radicals. GR: glutathione reductase; GPx: glutathione peroxidase; GSH: glutathione; GSSG: glutathione disulfide; ROS: reactive oxygen species.

Glutathione reductase is a critical enzyme that helps cells combat oxidative stress (Tanaka et al., 2002). The accumulation of reactive oxygen species (ROS) can lead to oxidative stress, which is detrimental to cellular components and a risk factor for the development of numerous diseases (Burton and Jauniaux, 2011; Sies, 2020). Therapeutic applications may arise from the inhibition of glutathione (GSH), a crucial antioxidant, due to the association of excessive oxidative stress with a multitude of diseases. A promising approach in the pursuit of targeted cancer therapy and antimicrobial strategies involves the inhibition of glutathione reductase (Figure 4) (Güller et al., 2021; Khatun et al., 2021). This approach exploits the dependency of cancerous cells and certain bacteria on the glutathione system for antioxidant defense (Khatun et al., 2021). By selectively compromising their ability to neutralize free radicals, inhibiting glutathione reductase can render cancer cells more susceptible to oxidative stress, potentially enhancing the effectiveness of conventional cancer treatments and overcoming drug resistance (Zhao et al., 2009). In bacterial infections, disrupting the bacterial antioxidant defense mechanisms through glutathione reductase inhibition may offer a means to sensitize pathogens to oxidative stress and improve the efficacy of antimicrobial treatments (Ren et al., 2020). This strategy, when carefully implemented, may exhibit synergistic effects with other therapeutic modalities, ultimately contributing to a more targeted and potent approach to combating cancer and bacterial infections (Staerck et al., 2017). Nonetheless, the development of selective inhibitors and a nuanced understanding of the specific cellular contexts are crucial considerations to mitigate potential adverse effects on normal cells and tissues.

**FIGURE 5:**
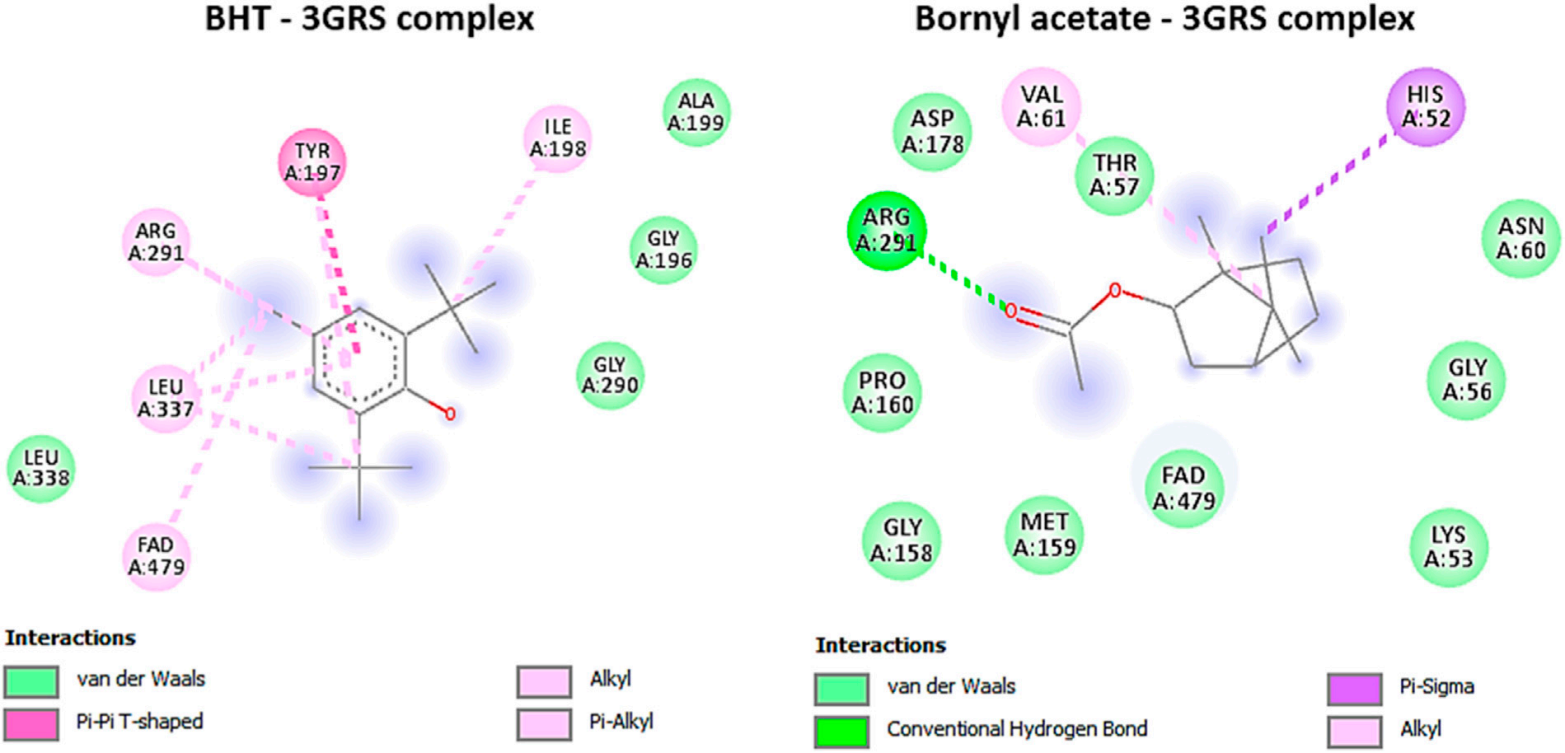
2D Molecular docking interactions of butylhydroxy toluene, and bornyl acetate, with glutathione reductase (PDB: 3GRS), (resolution: 1.54 Å), root mean square deviation (RMSD) < 1.

Our investigation revealed that bornyl acetate had a substantial binding affinity of −6.3 kcal/mol, which was similar to the synthetic antioxidant butylated hydroxytoluene (BHT) with a binding affinity of −6.3 kcal/mol (Table 3). Significantly, bornyl acetate generated just one hydrogen bond with an amino acid residue located inside the binding site pocket (ARG A:291), but BHT did not create any hydrogen bonds with the amino acid residues in the binding pocket (Figure 5).

#### 2.4.2 Interactions with dihydrofolate reductase (PDB: 4M6J): Antibacterial activity

**FIGURE 6:**
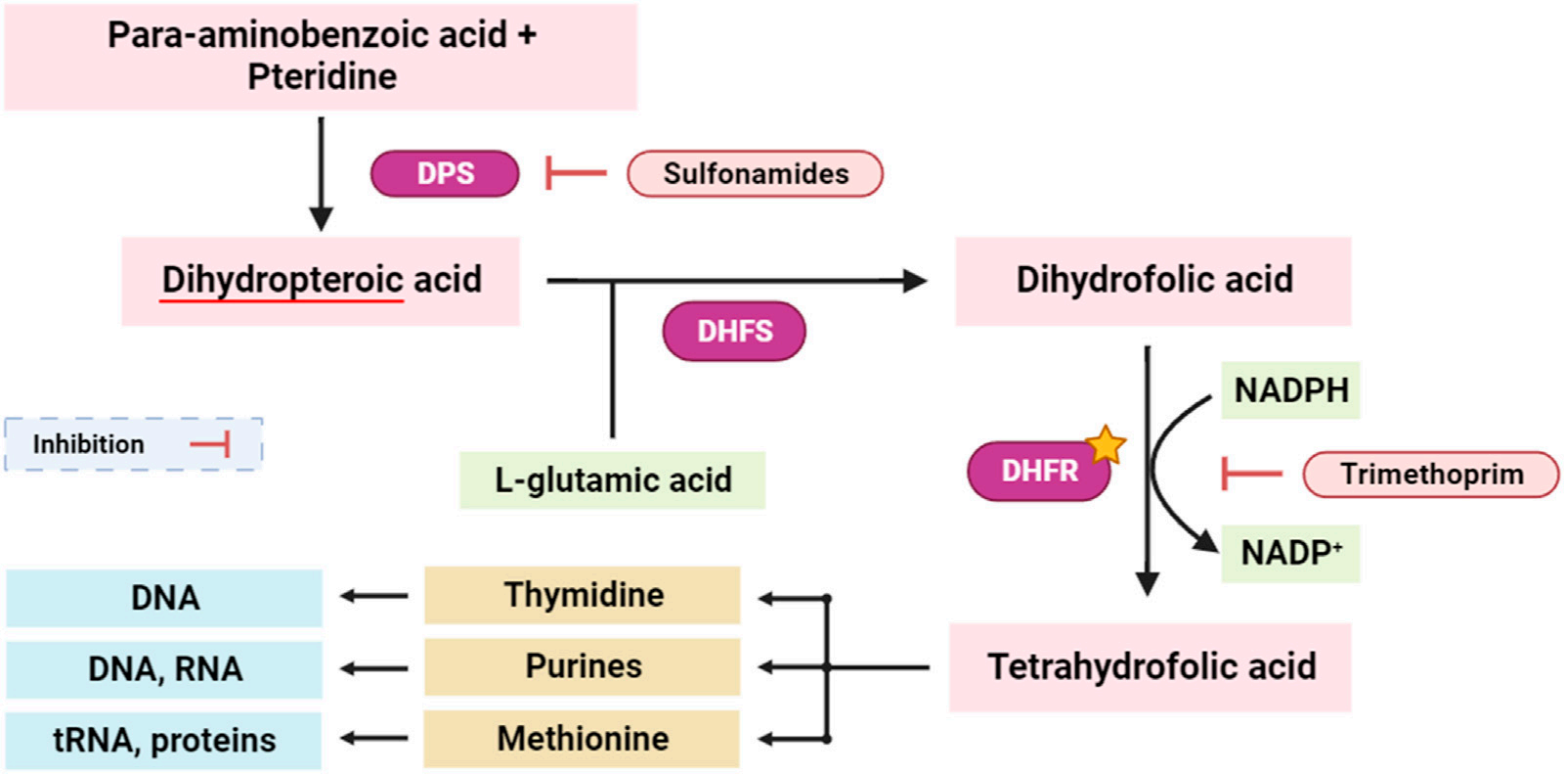
Simplified scheme of tetrahydrofolate synthesis pathway. DPS: dihydropteroate synthase; DHFS: dihydrofolate synthase; DHFR: dihydrofolate reductase. The yellow star indicates the potential targeted enzyme (DHFR).

Dihydrofolate reductase (DHFR) is an enzyme involved in the synthesis of tetrahydrofolate, which is required for the formation of purines, pyrimidines, and some amino acids (Andrews and Dyer, 2022). Inhibition of DHFR has been a key target for antibacterial, antifungal, anti-tuberculosis, and anticancer drug development. Concurrently, interference with the tetrahydrofolate synthesis pathway disrupts the synthesis of essential biomolecules, including purines, thymidine, and methionine. This dual-targeted approach has profound implications, as disruption of the tetrahydrofolate pathway critically impacts the availability of nucleotide precursors (purines and thymidine) and key methyl donors (methionine), pivotal for DNA replication, repair, and cellular methylation processes (Figure 6) (Chawla et al., 2021; Bhagat et al., 2022).

**FIGURE 7:**
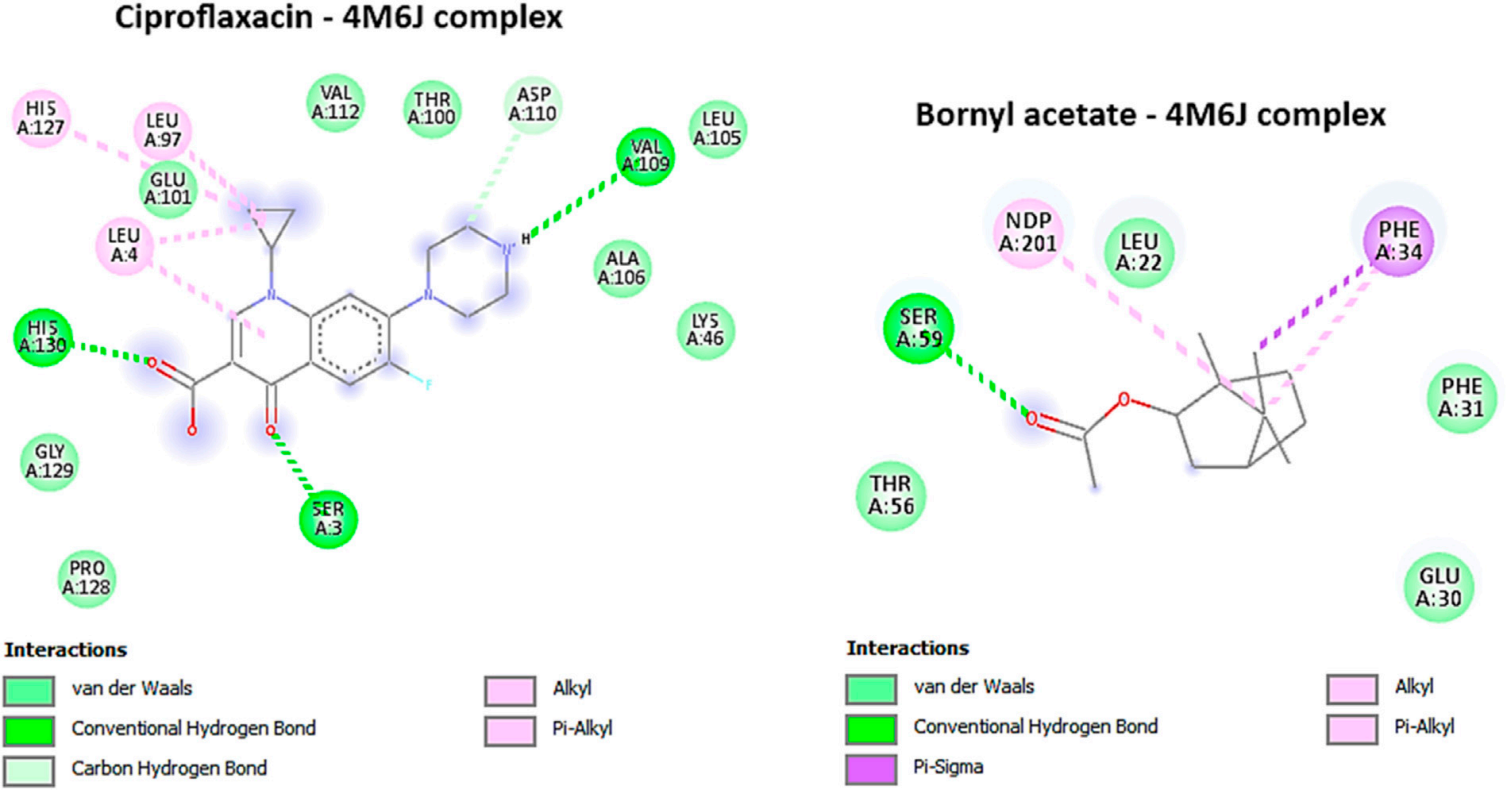
2D Molecular docking interactions of Ciproflaxacin, and Bornyl acetate, with dihydrofolate reductase (PDB: 4M6J). (resolution: 1.20 Å), root mean square deviation (RMSD) < 1.

The protein structure, PDB ID 4M6J, was obtained from the RCSB Protein Data Bank and depicts the crystallographic representation of DHFR (Dihydrofolate Reductase) from *S. aureus* (Bhabha et al., 2013). Given that *Staphylococcus aureus* is a virulent pathogenic bacterium responsible for a variety of infections, including skin and soft tissue maladies, bone infections, and others, this structural insight holds promise as a potential candidate for antibacterial therapeutic development (Bhabha et al., 2013). Targeting DHFR proves to be a strategically significant endeavor since inhibiting this enzyme disrupts the biosynthesis of tetrahydrofolate, ultimately culminating in the depletion of essential folate coenzymes vital for the synthesis of nucleic acids (comprising DNA and RNA) and specific amino acids (Wróbel et al., 2020). Consequently, DHFR emerges as an alluring focal point for the advancement of antibacterial drugs, holding the potential to impede bacterial growth and proliferation effectively (Bayazeed et al., 2022). In this study, bornyl acetate was found to have a potent docking score of −7 kcal/mol, similar to that of ciprofloxacin (a broad-spectrum antibiotic from the fluoroquinolones group), suggesting the potential of bornyl acetate as a potent antibacterial agent in the essential oil (Table 3). It was shown that ciprofloxacin displayed three hydrogen bonds with the amino acid residues from the binding pocket of the protein, namely, SER A: 3, VAL A: 109, and HIS A: 130. On the other hand, bornyl acetate demonstrated just one hydrogen bond with SER A: 59, which was located in the active site (Figure 7).

#### 2.4.3 Interactions with Cytochrome P450 alpha-sterol demethylase (CYP51, PDB: 1EA1): antifungal activity

**FIGURE 8:**
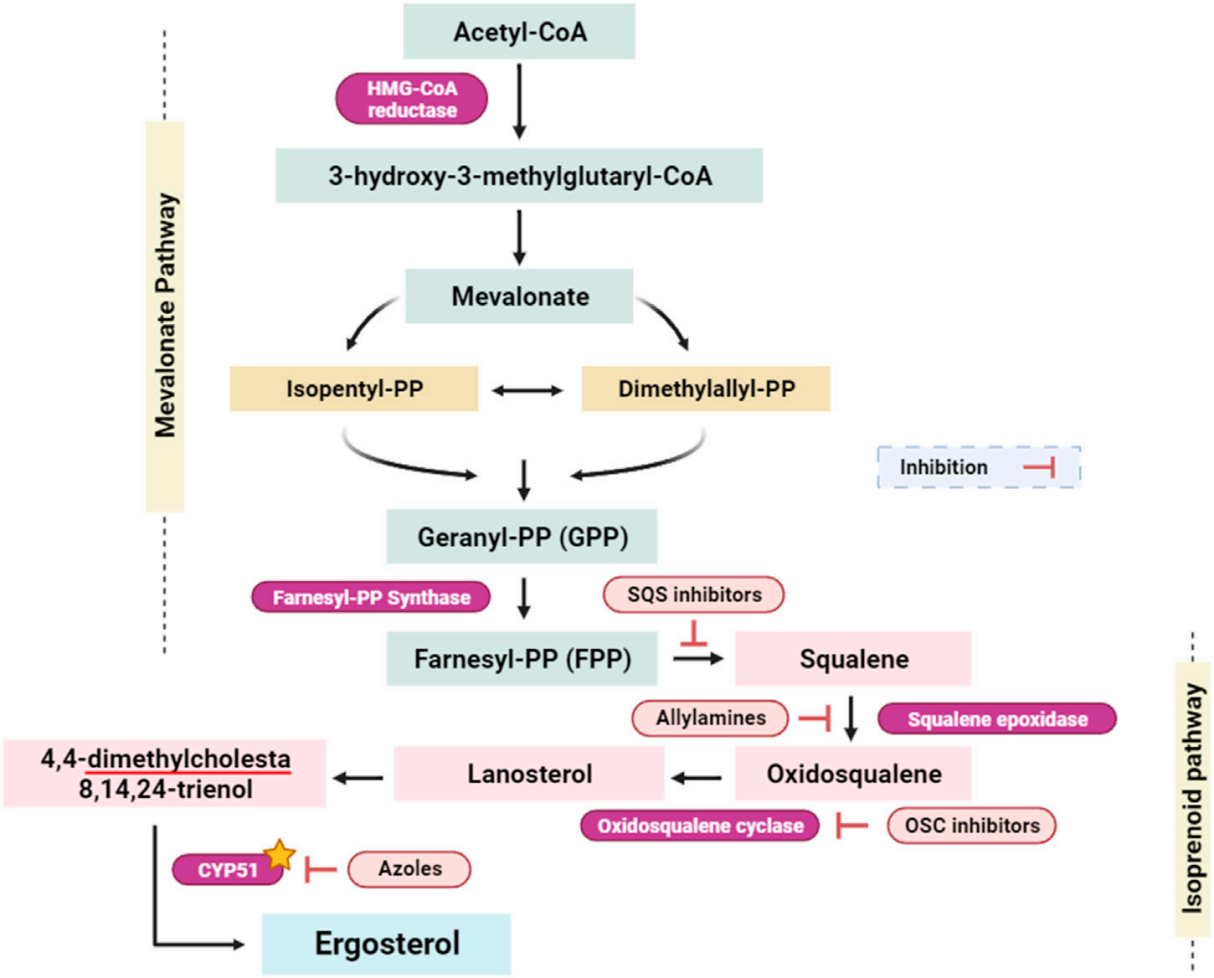
Simplified scheme of the ergosterol-implicated biosynthesis pathways (Raimondi et al., 2019; Prajapati et al., 2022). Isopentyl-PP: isopentyl pyrophosphate; Dimethylallyl-PP: dimethylallyl pyrophosphate; Geranyl-PP: geranyl pyrophosphate; Farnesyl-PP: farnesyl pyrophosphate; SQS inhibitors: squalene synthase inhibitors; OSC inhibitors: oxidosqualene cyclase inhibitors; CYP51: Cytochrome P450 alpha-sterol demethylase. Yellow star indicates the targeted enzyme within this study.

CYP51, known also lanosterol 14α-demethylase, is a crucial enzyme in the ergosterol biosynthesis pathway, which is exclusive to fungal cells (Parker et al., 2014). Ergosterol is an essential constituent of the fungal cell membrane, similar to cholesterol in human cells (Hu et al., 2017). Inhibiting CYP51 disrupts the process of ergosterol synthesis, resulting in a drop in ergosterol levels and the accumulation of toxic sterol intermediates (Figure 8). (Strushkevich et al., 2010; Hu et al., 2017). This disturbance jeopardizes the integrity and fluidity of the fungal cell membrane, making it more permeable and less functional. The inhibition of CYP51 affects a wide range of pathogenic fungi. Given the pivotal role of ergosterol as a foundational constituent within fungal cell membranes, the targeted inhibition of the enzyme lanosterol 14α-demethylase, or CYP51, emerges as a potent strategy with broad-spectrum antifungal efficacy (encompassing both yeasts and molds). CYP51 is an important enzyme in the ergosterol biosynthesis pathway, catalyzing the demethylation of lanosterol to produce ergosterol. Because ergosterol is essential for maintaining the structural integrity and functioning of fungal cell membranes, inhibiting its manufacture by CYP51 suppression has strong antifungal effects (Figure 8). Parker et al. (2014); Zhang et al. (2019). This broad-spectrum activity is essential for the development of antifungal agents that can combat different types of fungal infections. One of the advantages of targeting CYP51 is that ergosterol is not produced in human cells, which predominantly use cholesterol for membrane structure (Suárez et al., 2002; Lepesheva et al., 2008). This key difference in sterol biosynthesis between humans and fungi allows for the development of antifungal agents that selectively target fungal cells with minimal toxicity to the host.

**FIGURE 9:**
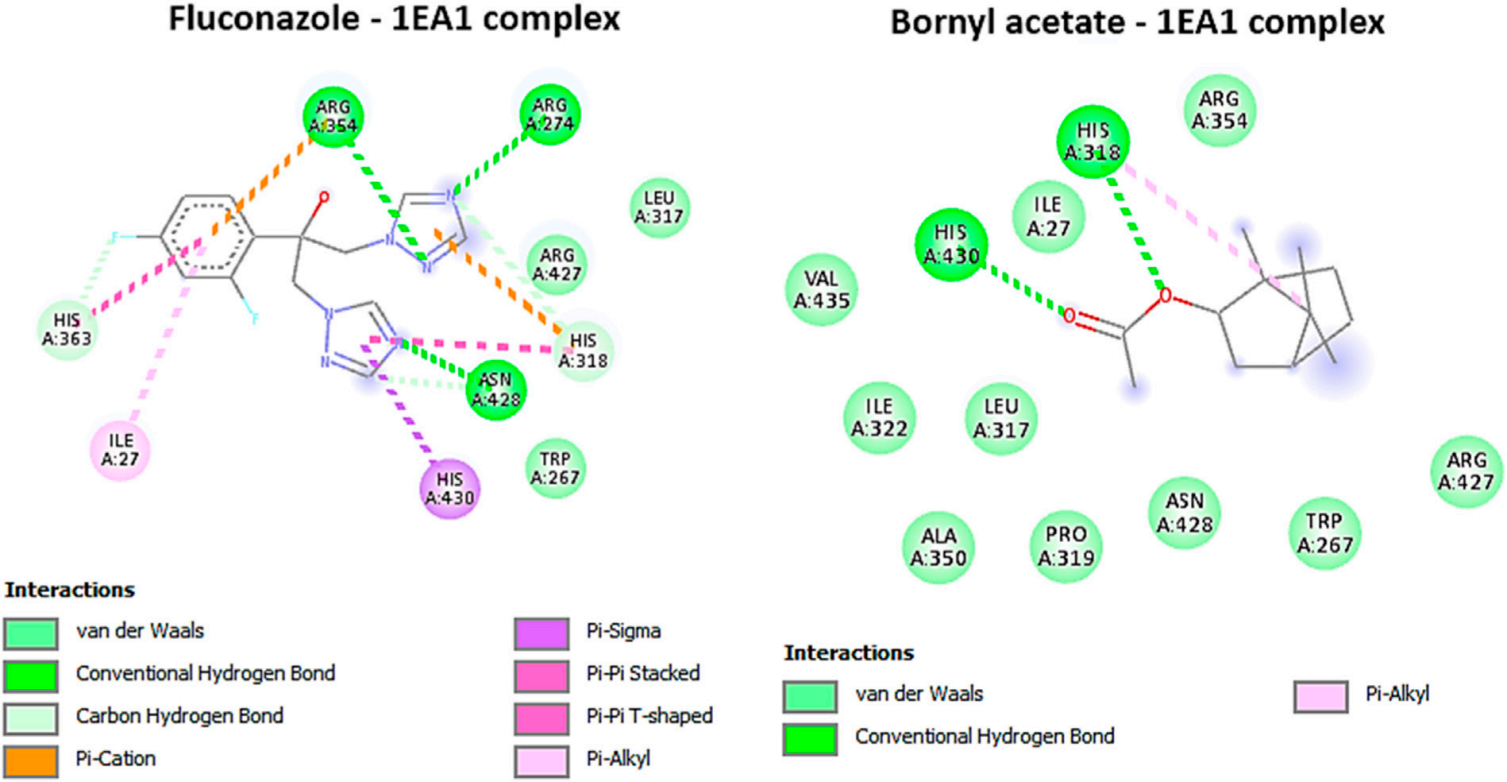
2D Molecular docking interactions of Fluconazole, and bornyl acetate, with Cytochrome P450 alpha-demethylase (PDB: 1EA1). (resolution: 2.21 Å), root mean square deviation (RMSD) < 1.

Based on the ongoing inquiry, it was discovered that bornyl acetate had a binding affinity of −5.8 kcal/mol, which closely resembled that of the antifungal drug fluconazole. This finding highlights the significance of bornyl acetate as a potential antifungal agent, as shown in Table 3. Bornyl acetate formed two hydrogen bonds with HIS A: 318 and HIS A: 430 at the binding site. On the other hand, fluconazole formed three hydrogen bonds with ARG A:274, ARG A:354, and ASN A: 428 (Figure 9).

#### 2.4.4 Interactions with 5-LOX (PDB: 1N8Q): anti-inflammatory activity

**FIGURE 10:**
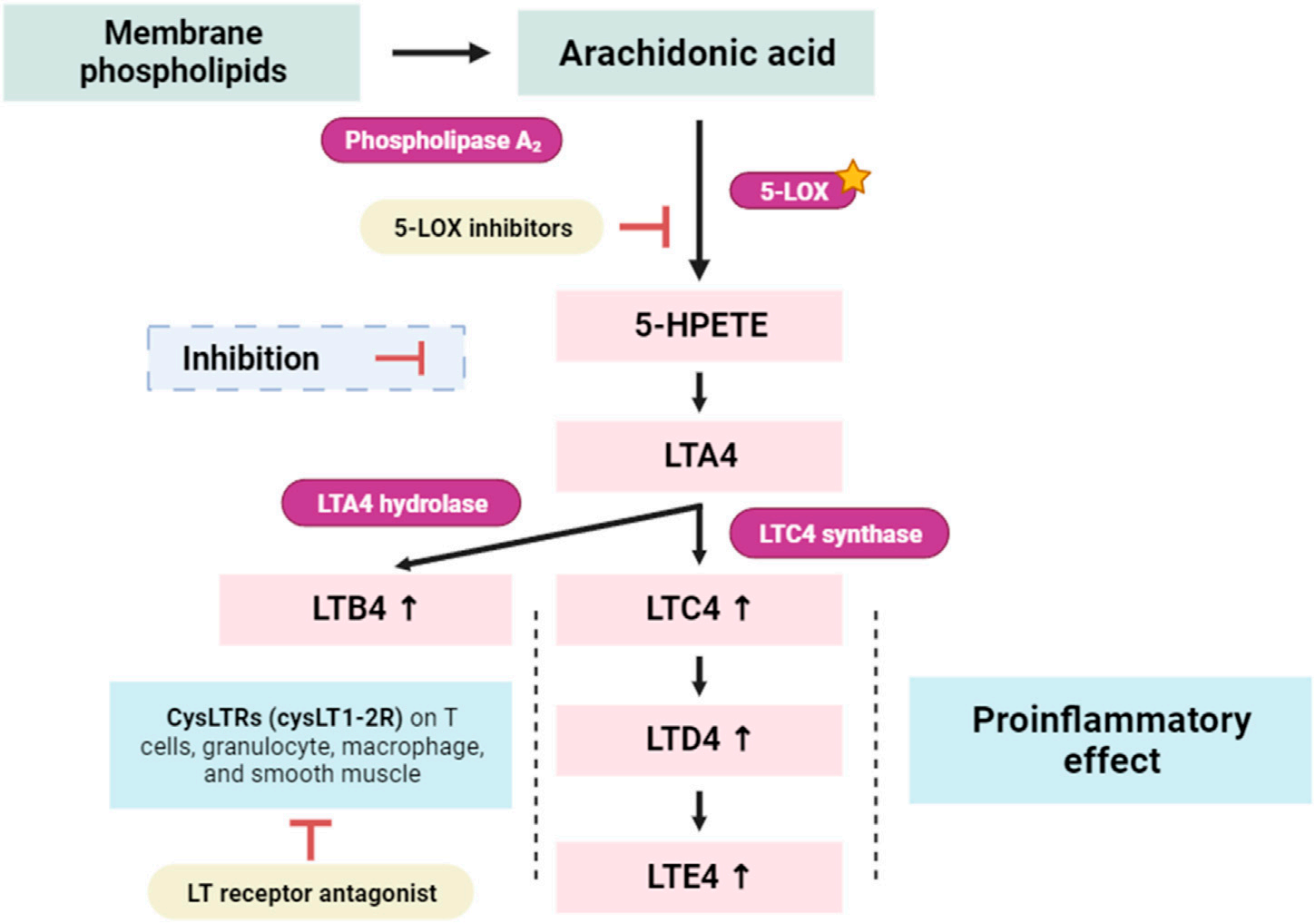
Simplified scheme of the conversion of arachidonic acid to 5-Hydroperoxyeicosatetraenoic acid (5-HPETE), and leukotrienes (LTs). 5-LOX: 5-lipoxygenase; LTA4, LTB4, LTC4, LTD4, and LTE4: Leukotrienes forms, known as cysteinyl leukotrienes; CysLTR1: LTA4 Cysteinyl leukotriene receptor 1 (Yamamoto, 1999).

Lipoxygenases (LOXs) are widely distributed in nature and are abundant in both plants and animals (Baysal and Demirdöven, 2007). These enzymes primarily act on polyunsaturated fatty acids (PUFA) containing cis double bonds. In animals, 20-carbon ascorbic acid (AA) is a prevalent substrate (Baysal and Demirdöven, 2007). LOX enzymes are named based on the specific carbon they oxygenate. Examples include 9-LOX and 13-LOX in plants, and 5-LOX, 12-LOX, and 15-LOX in animals (Brash, 1999). LOXs play a vital role in various biological functions by producing hydroperoxides, which are further transformed into important signaling molecules and biological mediators (Haeggström, 2018). On the other hand, negative outcomes are also possible with LOX-catalyzed reactions (Manju et al., 2018; Mahnashi et al., 2021). Leukotrienes (LTs) represent a crucial group of lipid mediators synthesized from arachidonic acid through the 5-lipoxygenase pathway (Figure 10). This biochemical cascade initiates with the conversion of arachidonic acid to leukotriene A4 (LTA4), subsequently undergoing enzymatic transformations to yield the bioactive molecules LTB4, LTC4, LTD4, and LTE4 (Yamamoto, 1999). Notably, these LTs play a pivotal role in diverse physiological processes and are particularly implicated in conditions such as allergic disorders (e.g., asthma and atopy), and systemic inflammatory diseases, including rheumatoid arthritis and cancer, as highlighted by (Henderson, 1994).

**FIGURE 11:**
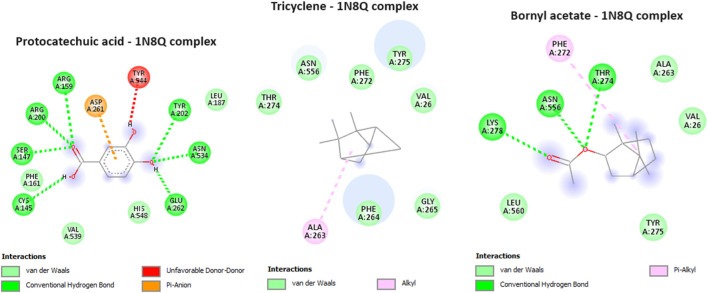
2D Molecular docking interactions of protocatechuic acid (native ligand of 5-LOX), tricyclene, and bornyl acetate, with 5-lipoxygenase (PDB: 1N8Q). (resolution: 2.10 Å), root mean square deviation (RMSD) < 1.

In our current investigation, we made a significant discovery regarding the binding interactions between tricyclene and bornyl acetate with the studied protein, indicating their strong inhibitory potential. With a score of −6.2 kcal/mol, tricyclene showed an excellent binding affinity, whereas bornyl acetate showed a very favorable value of −6.0 kcal/mol (Table 3). To put these findings in context, we compared them with the binding affinity of the native ligand, protocatechuic acid. Tricyclene’s interaction with the protein involved an alkyl interaction with a specific amino acid residue, ALA A: 263. In contrast, bornyl acetate established three typical hydrogen bonds with THR A: 274, LYS A: 278, and ASN A: 556, illustrating the variety of its binding interactions. Notably, when comparing these results to the native ligand, we observed that the native ligand engaged in seven hydrogen bonds, as illustrated in Figure 11. These findings reveal the potential of tricyclene and bornyl acetate as inhibitors of the studied protein, and the varied nature of their binding interactions underscores their promise as candidates for further exploration in the development of novel therapeutic agents.

#### 2.4.5 Interactions with epidermal growth factor receptor (PDB: 1XKK): anti-cancer activity

The epidermal growth factor receptor (EGFR), commonly referred to as ErbB1, is a protein located on the cell membrane that has a crucial function in regulating cell development and differentiation (Normanno et al., 2006). It has a role in several cellular activities, such as proliferation and survival (Zhang et al., 2017). In the context of breast cancer, EGFR has been a subject of significant interest and research for its potential anti-cancer implications. EGFR is often overexpressed in certain subtypes of breast cancer, particularly in triple-negative breast cancer (TNBC) (Nakai et al., 2016). This overexpression is associated with a more aggressive phenotype and poorer prognosis. Targeting EGFR can be a strategy to counteract this aggressive behavior (Nakai et al., 2016).

**FIGURE 12:**
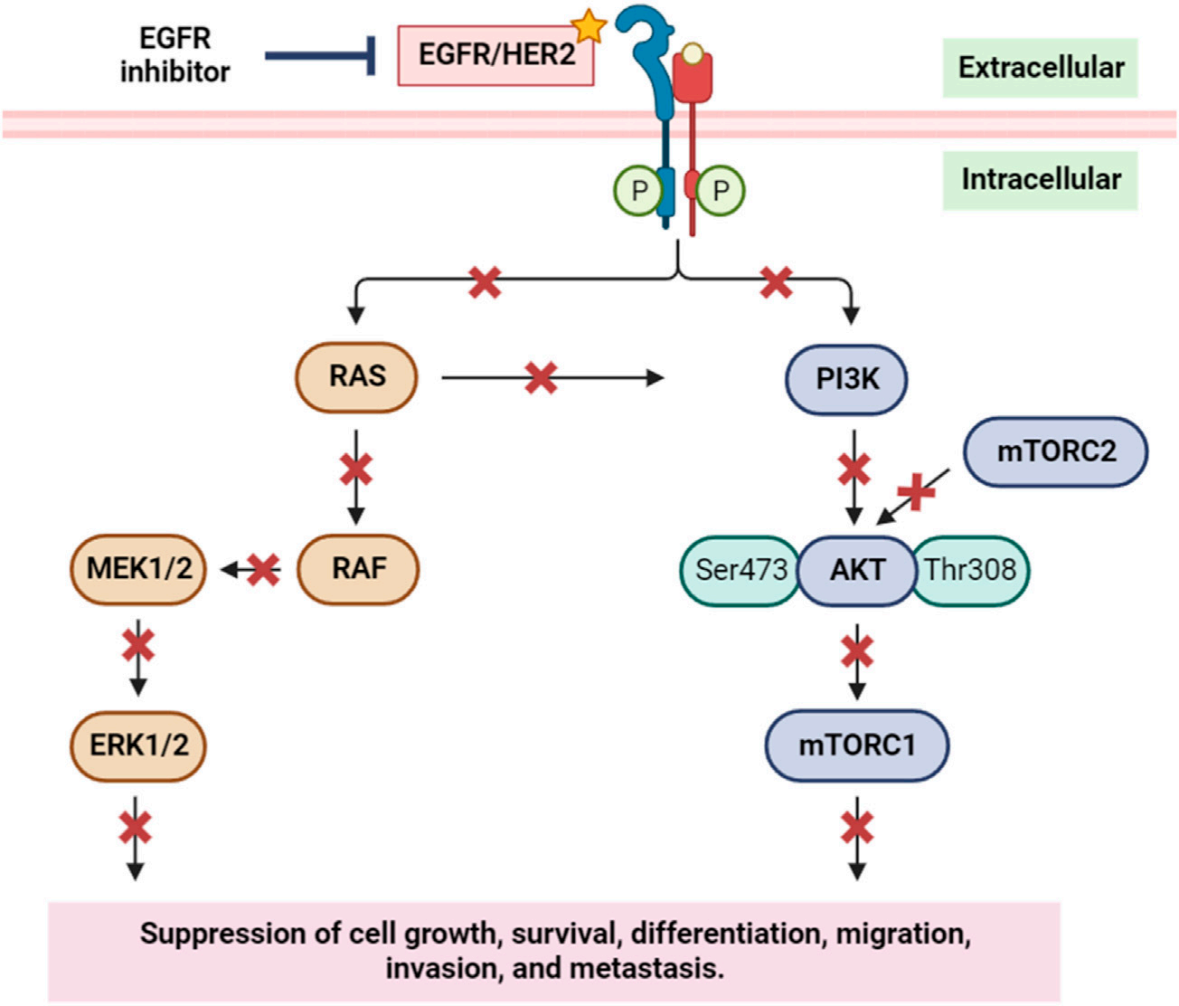
Simplified scheme of the main components of the EGFR/HER2 signaling pathway, and the inhibition of EGFR and its implication on cell growth, survival, migration, and metastasis. RAS: rat sarcoma; RAF: rapidly accelerated fibrosarcoma; MEK1/2: mitogen-activated protein kinase kinase 1 and 2; ERK1/2: extracellular signal-regulated kinase 1 and 2; PI3K: phosphoinositide 3-kinase; AKT: protein kinase B; mTORC1/2: mammalian target of rapamycin complex1/2. Yellow star indicates the targeted protein in the *in silico* study.

The EGFR/RAS/RAF signaling pathway is a crucial regulator of tumor cell survival and proliferation. Elevated expression of the EGFR is observed in various epithelial tumors, including non-small cell lung cancer, head and neck squamous cell carcinoma (HNSCC), colorectal cancer, and breast cancer (Herbst, 2004; Goerner et al., 2010). To counteract this, numerous anti-EGFR agents have been developed, showcasing significant anti-tumor activities in these cancer types (Bonner et al., 2006). EGFR’s pivotal role in initiating and progressing various human cancers, notably breast cancer, is well-established. Downstream of EGFR, the PI3K/AKT pathway assumes a critical role in regulating diverse cellular processes such as growth, survival, proliferation, and migration. Inhibiting EGFR not only impacts the EGFR/RAS/RAF signaling pathway but also disrupts the EGFR/PI3K/AKT/mTOR pathway (Figure 12) (Safdari et al., 2015). This dual inhibition suppresses cancer cells’ ability to grow, survive, differentiate, migrate, and metastasize, emphasizing the therapeutic potential of targeting EGFR in the context of cancer treatment.

**FIGURE 13:**
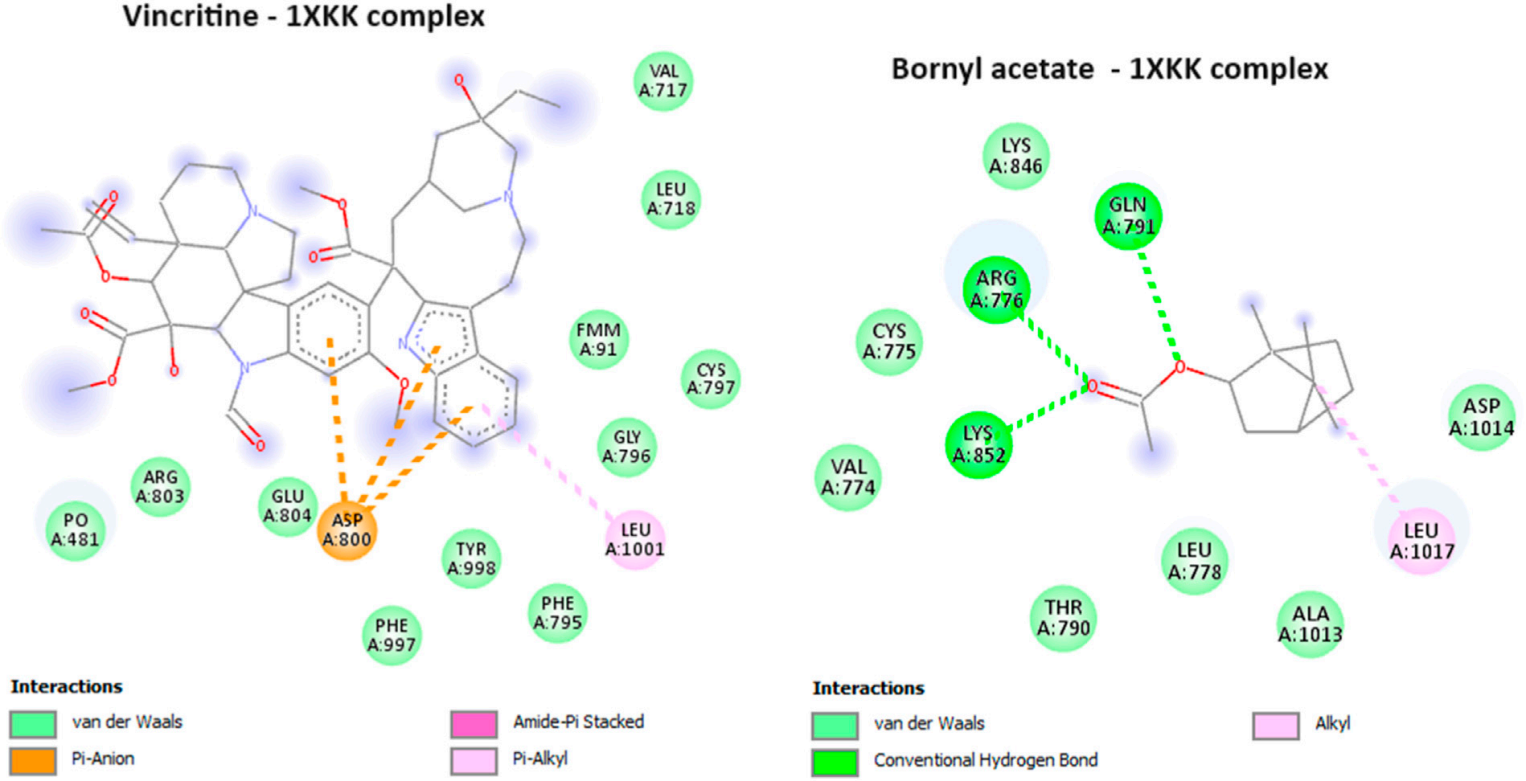
2D Molecular docking of vincristine (potent anti-cancer alkaloid), and bornyl acetate, with epidermal growth factor receptor (PDB: 1XKK). (resolution: 2.40 Å), root mean square deviation (RMSD) < 1.

During the course of our investigation, we identified that out of all the molecules analyzed, only one exhibited a noteworthy affinity towards the EGFR protein, as evidenced by its free binding energy of −5.9 kcal/mol (Table 3). On the other hand, the value of the widely recognized anti-cancer drug vincristine was marginally lower at −5.8 kcal/mol. After conducting an analysis of the interactions between these ligands and the protein, it was determined that bornyl acetate established three conventional hydrogen bonds with particular amino acid residues located at the active site: ARG A:776, GLN A:791, and LYS A:852 (Figure 13).

### 2.5 Experimental validation of the tested biological activities of CAEO

#### 2.5.1 Antioxidant activity

The antioxidant potential of *C. albidus* essential oil (CAEO) was assessed through various *in vitro* methods, with Table 4 providing a concise summary of the obtained results. CAEO displayed noteworthy capabilities in scavenging DPPH free radicals, as evidenced by a mean inhibitory concentration (IC_50_) of 153.92 ± 4.30 μg/mL, a value surpassing that of ascorbic acid, a recognized reference antioxidant, which exhibited an IC_50_ of 183 ± 3.54 μg/mL. Moreover, CAEO demonstrated a moderate antioxidant effect in the β-carotene bleaching assay, with a mean effective concentration (EC_50_) of 95.25 ± 3.75 μg/mL, underscoring its ability to safeguard beta-carotene from oxidation. Nonetheless, this value was higher than that of butylated hydroxytoluene (BHT), a synthetic antioxidant, which showcased an IC_50_ of 31.35 ± 3.20 μg/mL. In addition, CAEO exhibited moderate activity in the ABTS radical neutralization test, revealing an IC_50_ of 120.51 ± 3.33 μg/mL. The total antioxidant capacity (TAC) of CAEO was measured to be 458.25 ± 3.67 µg of ascorbic acid equivalent per mg of essential oil. In general, several species of Cistus have been discovered to exhibit significant antioxidant activities. For example, in a study conducted by Tomàs-Menor et al. (Tomás-Menor et al., 2013), hydroalcoholic extracts from diverse *Cistus* species demonstrated high antioxidant capacity in tests such as ferric ion reducing antioxidant power (FRAP), oxygen radical absorbance capacity (ORAC), Trolox equivalent antioxidant capacity (TEAC), and inhibition of lipid peroxidation (TBARS) Assay. Additionally, research by Amensour et al. (Amensour et al., 2010) on methanolic extracts of *Cistus ladaniferus* revealed significant antioxidant activity in in vitro assays, encompassing DPPH, ABTS, reducing power, metal chelation, and inhibition of the TBARS test. Furthermore, another investigation conducted by Dimcheva and Karsheva (2018), involving extracts from various parts of *Cistus incanus* demonstrated notable antioxidant activity through the DPPH assay.

**TABLE 4:** Free radical scavenging and antioxidant capacity of CAEO.

EO/Reference	DPPH scavenging capacity IC_50_ (µg/mL)	β-Carotene bleaching assay (µg/mL)	ABTS scavenging activity IC_50_ (µg/mL)	Total antioxidant capacity^a^
CAEO	153.92 ± 4.30	95.25 ± 3.75	120.51 ± 3.33	458.25 ± 3.67
Ascorbic acid (AA)	183.52 ± 3.54	—	23.54 ± 2.10	—
Butylated hydroxytoluene (BHT)	—	31.35 ± 3.20	—	—

^a^ TAC, expressed in µg of ascorbic acid equivalents per milligram of the essential oil.

#### 2.5.2 Antibacterial, and antifungal activities

In this study, the disc diffusion technique was used to measure inhibition zone diameters, and microdilution was performed to estimate the Minimum Inhibitory Concentration (MIC), Minimum Bactericidal Concentration (MBC), and Minimum Fungicidal Concentration (MFC). According to the reference Taibi et al. (2023a), essential oils are deemed active if they produce a microbial growth inhibition zone of 15 mm or greater. In the context of our study, the essential oil employed demonstrated considerable potent antibacterial effects against the examined bacterial strains, as detailed in the Results section (Tables 5, 6). The observed inhibition zones ranged from 11 to 25 mm. As per the findings in Tables 5, 6, the results for inhibition zone diameters revealed that the essential oil CAEO exhibited important activity against *M. luteus*, *L. monocytogenes*, and *G. candidum*, with inhibition zone diameters falling within the range of 17–25 mm. However, it showed modest activity against *R. glutinis*, *E. coli* and *S. aureus* with an inhibition zone diameter of 15, 14, and 11 mm, respectively. The data in Tables 5, 6 reveals that CAEO exhibited notable inhibitory potential against all examined strains. The Minimum Inhibitory Concentration (MIC) values were found to be 8% for *Escherichia coli*, *S. aureus*, *Listeria monocytogenes*, and *Geotrichum candidum*, while it was 1% for *Micrococcus luteus*. However, *Rhodotorula glutinis* exhibited a MIC value surpassing 16%. In the case of the yeast *R. glutinis*, CAEO exhibited modest inhibitory and fungicidal activity, with a MIC, and MFC values exceeding 16%. For the fungus *G. candidum*, CAEO displayed effective antifungal activity, with a MIC of 8% and an MFC of 8%.

**TABLE 5:** Evaluation of the minimum inhibitory concentrations and the bactericidal concentrations of CAEO.

Gram	Bacterial strains	CAEO			Gentamicin (1 mg/mL)
		MIC (%)	MBC (%)	IZ (mm)	IZ (mm)
G-	*E. coli*	8	16	11 ± 0.50	21.50 ± 0.50
G+	*M. luteus*	1	2	25 ± 1.00	22.50 ± 1.00
G+	*S. aureus*	8	16	14 ± 0.33	18.50 ± 1.00
G+	*L. monocytogenes*	8	16	23 ± 0.25	19 ± 0.33

**TABLE 6:** Evaluation of the minimum inhibitory concentrations and the fungicidal concentrations of CAEO.

Type	Fungal strains	CAEO			Cycloheximide (1 mg/mL)
		MIC (%)	MFC (%)	IZ (mm)	IZ (mm)
Yeast	*R. glutinis*	>16	16	15 ± 0.50	21.00 ± 0.50
Fungi	*G. candidum*	8	8	17 ± 1.00	23.50 ± 0.33

Although the genus *Cistus* has been intensively investigated in terms of its antimicrobial properties, the study of the essential oil of the antimicrobial potential of CAEO remains scarce. These outcomes can be related to the chemical compounds of CAEO that showed a high content of monoterpenes. Indeed, several reports stated that monoterpenes and sesquiterpenes are prominent compounds with antifungal and antibacterial activities (Pereira et al., 2017; Silva et al., 2021). The antibacterial action of essential oils and their monoterpenoid components has typically been attributed to toxic effects on membrane structure and function. Indeed, due to their lipophilic nature, monoterpenes will preferentially distribute from an aqueous phase to membrane structures. As a result, the membrane enlarges, and becomes more fluid and permeable, proteins imbedded in the membrane are disturbed, respiration is inhibited, and ion transport pathways are changed (Trombetta et al., 2005).

#### 2.5.3 Anti-inflammatory activity

**FIGURE 14:**
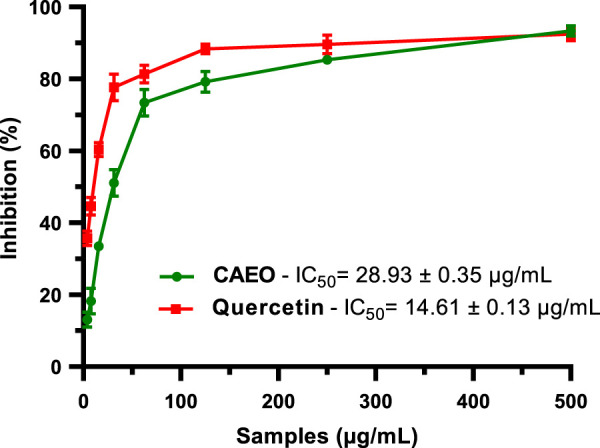
The percentage of LOX inhibition for the essential oil of *Cistus albidus*, as well as quercetin which was employed as a positive control. The mean results of three distinct studies were computed and expressed as the mean value plus or minus the standard deviation (Mean ± SD).

Figure 14 shows the findings of the lipoxygenase inhibition experiment, which examined anti-inflammatory activity. The essential oil from C. albidus inhibited lipoxygenase significantly, with an IC50 value of 28.39 ± 0.35 μg/mL. This shows that CAEO has a significant ability to inhibit the activity of lipoxygenase, a crucial enzyme implicated in inflammatory processes (Mahnashi et al., 2021). In contrast, quercetin, a well-known reference drug noted for its inhibitory characteristics, demonstrated significant lipoxygenase inhibition with an IC50 value of 14.61 ± 0.13 μg/mL. Both samples clearly had significant anti-inflammatory potential; nevertheless, there were apparent variations in their capacity to inhibit lipoxygenase, with quercetin showing higher efficacy in this aspect. CAEO’s anti-inflammatory action is mostly due to the presence of bioactive compounds. Essential oils are typically composed of volatile chemicals collected from plants, and several studies have shown that many of these molecules have anti-inflammatory characteristics (Sharopov et al., 2015; El Hachlafi et al., 2023b; Elsayed et al., 2023).

One of the major bioactive compounds in this essential oil, α-pinene, is responsible for CAEO’s anti-inflammatory activity. It can inhibit inflammatory responses by acting on different biological pathways (Rufino et al., 2014; Kim et al., 2015). Several researchers suggest that bornyl acetate, the major compound of this essential oil may have anti-inflammatory properties by inhibiting certain mechanisms of inflammation (Yang et al., 2014; Lee et al., 2023; Zhao et al., 2023). These findings indicate that CAEO could be a promising candidate among naturally-derived anti-inflammatory agents.

#### 2.5.4 Anticancer activity

**FIGURE 15:**
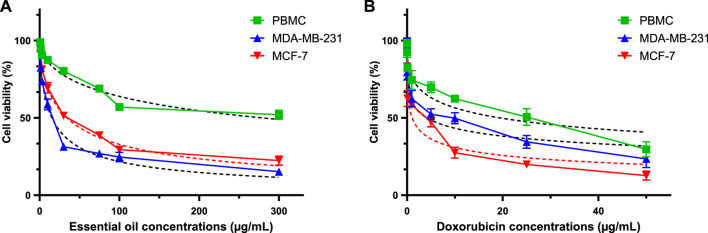
Using the MTT test, evaluation of cell viability in MCF-7, MDA-MB-231, and PBMC cells treated with CAEO **(A)** and doxorubicin [positive control, **(B)**] for 72 h.

Table 7 presents a thorough evaluation of the Selectivity Index (SI) and IC50 values for CAEO on various human breast cancer cell lines, including MCF-7 and MDA-MB-231, as well as on peripheral blood mononuclear cells (PBMC), which are a stand-in for healthy, human cells. CAEO has promising anti-cancer effects that are dose-dependent (Figure 15.), as indicated by varying IC_50_ values across the tested cell lines, for MCF-7, CAEO yields an IC50 value of 29.69 ± 3.15 μg/mL, while for MDA-MB-231, which is known for its aggressive characteristics, experiences a substantial inhibitory effect from CAEO, with an IC_50_ value of 12.41 ± 3.60 μg/mL. Contrasting with these cancer cell lines, PBMC, representing normal, healthy cells, exhibits a notably higher IC_50_ value of 209.83 ± 6.71 μg/mL when exposed to CAEO, underscoring the relative safety of the essential oil on healthy cells. To gauge the selectivity of CAEO in targeting cancer cells while sparing healthy ones, the Selectivity Index (SI) was calculated. This index is derived from the ratio of the IC_50_ values of PBMC to the tumor cells, with a higher SI indicating a heightened selectivity for cancer cells. For CAEO, MCF-7 demonstrates an SI of 7.067, indicating a considerable level of selectivity for this particular breast cancer cell line. In the case of MDA-MB-231, the SI notably escalates to 16.908, emphasizing the exceptional selectivity of CAEO for this specific subtype of breast cancer. For comparative purposes, Doxorubicin, a widely employed chemotherapeutic agent, exhibits robust anticancer efficacy with IC_50_ values for MCF-7 and MDA-MB-231 at 1.08 ± 0.13 μg/mL and 4.159 ± 0.66 μg/mL, respectively. The calculated SI values for Doxorubicin are 17.95 for MCF-7 and 4.662 for MDA-MB-231. These results collectively underscore the considerable promise of CAEO as an agent selectively targeting breast cancer cells, with a pronounced preference for the highly aggressive MDA-MB-231 cell line. Importantly, the SI values emphasize the relative safety of CAEO in relation to healthy PBMC, a pivotal consideration in the development of novel anticancer therapeutics.

**TABLE 7:** Assessment of the selectivity indexes and IC_50_ levels for CAEO on various human breast cancer cell lines (MCF-7, and MDA-MB-231).

Treatments	IC_50_ value ±SD (µg/mL)^a^			Selectivity index (SI)	
	MCF-7	MDA-MB-231	PBMC	MCF-7	MDA-MB-231
CAEO	29.69 ± 3.15	12.41 ± 3.60	209.83 ± 6.71	7.067	16.908
Doxorubicin	1.08 ± 0.13	4.159 ± 0.66	19.39 ± 2.55	17.95	4.662

^a^ The mean values of three separate experiments were calculated and presented as means with standard deviations.

The selectivity index was calculated as the ratio of the IC_50_ values of PBMC and tumor cells.

## 3 Materials and methods

### 3.1 Plant material and essential oil preparation

The aboveground portion of the plant under investigation, known as grey-leaved cistus (Cistus albidus L.), was gathered in the vicinity of “Ifrane” in the Middle Atlas area of Northern Morocco (33°32′09.4″N 5°08′23.3″W) during September 2022. The botanical identification was performed at the Department of Biology of the Faculty of Sciences, University Mohammed the first (Oujda, Morocco), and a voucher specimen number CLP-002 was later assigned. The aerial part of the plant was desiccated in a confined area until it attained a uniform weight, usually requiring around 6–7 days. Before hydrodistillation, the thoroughly dried samples were crushed into a fine powder. A revised Clevenger apparatus was used to conduct hydrodistillation of 100 g of plant material in 400 mL of water until the essential oil concentration reached a consistent level, which required around 2–3 h.

After the extraction process, anhydrous sodium sulfate was used to remove any leftover water. The extraction yields are computed using the following formula:

$$\text{Yield }\left(\%\right)=\left(\frac{M_{\text{extract}}}{M_{\text{sample}}}\right)\times 100$$

M-extract represents the quantity of oil in grams, while M-sample represents the mass of the plant sample in grams. Finally, prior to analysis, the essential oil was stored in a hermetically sealed glass container in a refrigerator at a temperature of 4°C.

### 3.2 Qualitative and semi-quantitative analysis of CAEO volatile compounds by GC/MS

The identification and quantification of volatile components of a complex mixture were carried out with a Gas chromatography (GC) technique coupled to a mass spectrometer type Shimadzu GC/MS-QP 2010 series (Loukili et al., 2023). The sample is vaporized and then injected with a split/splitless injector into the BP-X25 capillary column (LxDI: 30 m × 0.25 mm), packed with an apolar stationary phase material (95% dimethylpolysiloxane/5% phenyl). The helium is used as a carrier gas with a 3 mL/min flow rate. The temperatures for injection, ion source, and interface were adjusted to 250°C. The temperature profile for the column oven consisted of an initial hold at 50°C for 1 min, followed by a ramping up to 250°C at a rate of 10°C per minute, also for 1 min. The given components were subjected to ionization utilizing the electron impact (EI) mode at an energy of 70 electron volts (eV). The mass range that was scanned ranged from 40 to 300 m/z. The individual constituents are then identified and measured by a detector mass spectrometer. Ultimately, the sample components exhibit distinct interactions with the stationary phase while traversing the column, resulting in their separation according to their specific chemical characteristics. Compounds were recognized by the comparison of their retention durations with legitimate standards and their mass spectrum fragmentation patterns with those present in databases or kept on the National Institute of Standards and Technology (NIST). The data collection and processing were performed using LabSolutions (version 2.5).

### 3.3 Drug-likeness, ADME, the prediction of the toxicity analysis (Pro-Tox II)

Absorption, Distribution, Metabolism, and Excretion are important determinants of the pharmacokinetic characteristics of a substance, encompassing its processes of absorption, distribution, metabolism, and elimination from the body. Computational techniques are utilized to forecast the ADME characteristics of compounds, including their capacity to pass through cell membranes, their attraction to transporters and enzymes involved in drug absorption and elimination, and their metabolic stability. To evaluate the compounds, we utilized ADME webservers like SwissADME and pkCSM (Pires et al., 2015; Daina et al., 2017). These webservers helped us analyze the physicochemical properties, drug similarity, and pharmacokinetic properties of the compounds. The Pro-Tox II online tool (https://tox-new.charite.de/protox II/viewed on 21 March 2023) was utilized to assess toxicity levels (Banerjee et al., 2018). This online application utilizes a statistical algorithm to compare the chemical structure of a substance with an extensive database of recognized dangerous chemicals. It then forecasts the probability of the compound inducing toxicity or unfavorable effects in humans or other creatures. This tool offers data on the LD_50_ values, toxicity class, and toxicological endpoints, such as hepatotoxicity, carcinogenicity, immunotoxicity, mutagenicity, and cytotoxicity. The methodology and tools provide useful insights into the possible therapeutic uses and toxicity hazards of CAEO compounds.

### 3.4 Molecular docking Protocol

#### 3.4.1 Ligand and protein preparation

The specific phytochemicals present in CAEO were obtained from PubChem [source: https://pubchem.ncbi.nlm.nih.gov/; accessed on 10 September 2023]. The SDF format was used to obtain various standard drugs, such as the antioxidant BHT (PubChem CID: 31404), antibacterial and antifungal agents like ciprofloxacin (PubChem CID: 2764) and fluconazole (PubChem CID: 3365), the native ligand of Lipoxygenase, protocatechuic acid (PubChem CID: 72), and the well-known anticancer agent, vincristine (PubChem CID: 5978). Afterwards, these ligands are imported into Discovery Studio version 4.5 in order to generate a library of ligands in PDB format, using the matching PubChem CIDs. In order to improve the precision of molecular interactions using phy-toconstituents and standard ligands, the Pm6 semi-empirical technique was used. This method, renowned for its accuracy in predicting molecular geometries and energies, was employed as described by (Bikadi and Hazai, 2009).

We utilized a computational docking approach to predict the potential antioxidant, antibacterial, antifungal, anti-inflammatory, and cytotoxic properties of the identified compounds. Based on previously published research, we selected specific proteins to interact with: Glutathione reductase (PDB ID: 3GRS) for antioxidant properties (Rashid et al., 2023), dihy-drofolate reductase (DHFR) enzyme (PDB ID: 4M6J) for antibacterial effects (Khatun et al., 2021), Cytochrome P450 alpha-sterol demethylase (PDB ID: 1EA1) (Taibi et al., 2023a), 5-Lipoxygenase (PDB ID: 1N8Q) for anti-inflammatory activity (Tomy et al., 2018), epidermal growth factor receptor (PDB ID: 1XKK) for cytotoxicity (Jannat et al., 2022). The three-dimensional crystal structures of these chosen proteins were retrieved in PDB format from the RCSB protein data bank (source: https://www.rcsb.org; accessed 10 September 2022).

PyMoL 2.3 was used to streamline all macromolecules, including water molecules and unnecessary protein residues, as part of the preparation of proteins for molecular docking. Non-polar hydrogen atoms and Kollmann charges were then carefully added to finish and polish the purified protein structures in order to guarantee structural integrity (Cosconati et al., 2010). Energy optimisation using the Swiss PDB viewer—a tool well-known for its capacity in energy minimization, as reported by (Guex and Peitsch, 1997)—was the next step toward further development. Finally, the finalised macromolecules were carefully stored in PDB format to enable further detailed analyses and molecular docking studies.

#### 3.4.2 Ligand-protein interaction

The molecular docking approach was employed to predict the probable binding patterns and affinities of isolated plant metabolites with specific target biomolecules (Elbouzidi et al., 2022). During this computational interaction process, a semi-flexible modeling approach was used, and it was executed with the widely employed PyRx AutoDock Vina molecular docking software. The target proteins were prepared and labeled as macromolecules within PyRx (Pawar and Rohane, 2021). The 3D conformers of all ligands, initially in SDF format, were introduced into PyRx and energetically optimized. Subsequently, they were converted to pdbqt format within the PyRx AutoDock Vina software using the Open Babel tool, and the most optimal hit was selected (O’Boyle et al., 2011).

Grid boxes were established, with active binding sites for the proteins centered and mapped. For instance, the grid box mapping for the proteins was set at coordinates in Supplementary Table S1. All other docking parameters were retained at their default settings, with AutoDock Vina version 1.1.2 being used for the docking process. The outcomes of the docking analysis were projected, and the results, along with the docked macromolecules and ligands, were exported in pdbqt format as output files. These files for ligands and the macromolecule were merged and saved in PDB format for further examination using the PyMol software. Finally, 3D and 2D visualizations were generated using Discovery Studio Visualizer (version 4.6).

### 3.5 Non-enzymatic antioxidant assays

#### 3.5.1 2,2-Diphenyl-1-Picrylhydrazyl (DPPH) scavenging assay

The essential oil’s ability to scavenge DPPH radicals was according to the method described in (Zrouri et al., 2021; Al-Mijalli et al., 2023; Haddou et al., 2023), with slight modifications. Each concentration was tested three times. A 0.1 mM DPPH (2,2-diphenyl-1-picrylhydrazyl) solution was prepared, and various essential oil concentrations (0.05–1 mg/mL) were used. Ascorbic acid was employed as a positive control (Ouahabi et al., 2023).

#### 3.5.2 β-Carotene bleaching assay

To evaluate the antioxidant potential of CAEO, we employed the β-carotene bleaching assay, following the procedure outlined by Elbouzidi et *al*. (Elbouzidi et al., 2023). A series of CAEO concentrations, ranging from 0.01 to 10 mg/mL, was used. This assay was used to gauge CAEO’s capacity to inhibit β-carotene bleaching, thus assessing its antioxidative properties.

#### 3.5.3 ABTS scavenging activity assay

The radical scavenging potential of the test samples against the ABTS (2,2′-azinobis-3-ethylbenzothiazoline-6-sulphonate) radical cation was evaluated in accordance with the methodology outlined by Re et al. (1999), albeit with slight modifications (Elbouzidi et al., 2023). Ascorbic acid was employed as a reference standard, the IC_50_ (mg/L) for the radical scavenging capacity of CAEO was measured across concentrations spanning from 0.01 to 1 mg/mL. All measurements were conducted in triplicate, and the antioxidant activity data were presented as means ± SD from the triplicate measurements.

#### 3.5.4 Total antioxidant capacity (TAC)

Total antioxidant activity measures how well CAEO can protect the body from damage caused by harmful substances called free radicals (Chaudhary et al., 2015). We measured this using a method called the phosphorus-molybdenum technique, similar to a method used in a study by Elbouzidi et al. (2023). A standard curve was constructed based on vitamin C, and the findings were subsequently presented in terms of vitamin C equivalents (Prieto et al., 1999).

### 3.6 Anti-inflammatory activity

The anti-inflammatory potential of each sample was assessed *in vitro* by measuring its ability to inhibit soybean lipoxygenase using the FOX test (Sidiropoulou et al., 2022). The FOX test relies on the development of a reddish-brown complex between xylenol orange and Fe^3+^ under acidic conditions, which are produced by the hydroperoxides generated during the oxidation of linoleic acid by soybean lipoxygenase. Reagent quantities for the spectrophotometric analysis were modified according to the approach described by Ondua et al. (2019), as well as the concentration range. The CAEO samples were first diluted in dimethyl sulfoxide (DMSO) and then further diluted in Tris–HCl buffer (50 mM, pH 7.4) to attain a final concentration of 0.5 mg/mL. Quercetin was used as the positive control. Both CAEO and quercetin were evaluated across a concentration range spanning from 500 to 3.9 μg/mL. Afterwards, 200 μL of the essential oil (or quercetin) was mixed with Tris–HCl buffer (200 μL), and 400 μL of 5-LOX enzyme (Sig-ma-Aldrich, Taufkirchen, Germany), dissolved in ice-cold Tris–HCl buffer, was added to reach a final concentration of 0.2 U/mL. The solution was allowed to undergo incubation at ambient temperature for a duration of 5 min. Subsequently, 400 μL of linoleic acid, acting as the enzyme substrate, was added to the test mixture, resulting in a final concentration of 140 μM. The resulting mixture was then kept in the dark at room temperature for 20 min. Finally, 1,000 μL of the freshly prepared FOX reagent, comprising xylenol orange at 100 μM, FeSO_4_ at 100 μM, and H_2_SO_4_ at 30 mM in MeOH(aq) 90%, was added to the tubes, and the mixture was incubated for 30 min. The negative control contained a blend of DMSO and Tris–HCl buffer instead of CAEO, while in the blank samples, linoleic acid was added to the designated tubes just before the absorbance measurement at 560 nm, following the third incubation period. EO sample was paired with its negative control and blank. The inhibitory activity of each sample was calculated using the formula:

$$\%\text{inhibition}=\left[\left(\frac{\text{Ac}-\text{As}}{\text{Ac}}\right)\times 100\right]$$

Where Ac represents the absorbance of the negative control, and As represents the absorbance of the EO sample or quercetin after subtracting the absorbance of the corresponding blank. The results are expressed as the mean of three measurements ±SD. IC_50_ was determined from the plots.

### 3.7 Antibacterial activity

#### 3.7.1 Microorganisms

One Gram-negative bacteria, *E. coli* (ATCC 11775), as well as three Gram-positive bacteria, *M. luteus* (ATCC 10240), *S. aureus* (ATCC 6538), and *L. monocytogenes* (ATCC 19114), were selected for this study. Antifungal activity was tested on *G. candidum* and *R. glutinis*. These strains come from pure cultures available at the Microbial Biotechnology Laboratory, Faculty of Science, Oujda, Morocco.

#### 3.7.2 Disk diffusion method

We employed the suitable agar diffusion method to evaluate the antibacterial activity. We employed Mueller-Hinton agar (MHA) to culture bacteria and potato dextrose agar (PDA) to culture fungus (Elbouzidi et al., 2023; Haddou et al., 2023). The bacterial strains were introduced into Petri dishes containing agar medium using this technique. Filter paper discs with a diameter of 6 mm, which had been sterilized, were impregnated with 15 µL of essential oils. Gentamicin and cycloheximide were employed (at 1 mg/mL) as positive controls against bacteria and fungi, respectively; whereas DMSO was utilized as a negative control. The samples, including gentamicin, cycloheximide (positive controls), and DMSO (negative control), were placed on the agar surface using forceps that had been sterilized with a flame. The disks were then gently pressed down to establish proper contact with the surface. Following a 30-min pre-diffusion period at ambient temperature, the Petri plates were placed in an incubator set at 37°C for 18 h. The sizes of the growth zones that were prevented from growing were subsequently measured in millimeters (mm).

#### 3.7.3 Determination of MIC

The minimum inhibitory concentration (MIC) was measured using the resazurin reagent revelation method (Kandsi et al., 2022; Taibi et al., 2023a; Loukili et al., 2023). MIC refers to the lowest concentration that effectively inhibits bacterial growth following a 24-h incubation. The visual observation of the presence or absence of red color was used to determine the MIC.

#### 3.7.4 Determination of MBC and MFC

In order to ascertain the minimum bactericidal concentration (MBC), a 3 µL sample was extracted from the negative control wells and transferred to a nutrient-rich growth medium known as MHA. The sample was then incubated at a temperature of 37°C for 24 h. The term Minimum Bactericidal Concentration (MBC) was later coined to denote the lowest concentration of essential oil that does not lead to observable bacterial growth (Taibi et al., 2023a). In addition, the Minimum Fungicidal Concentration (MFC) of yeast was evaluated by extracting 3 µL from wells that showed no growth., transferring them onto a PDA medium, and allowing them to incubate at a temperature of 25°C for 48 h. The identical procedure was employed for molds, with the exception that the period of incubation was prolonged to 72 h at a temperature of 25°C. Following this period of incubation, the minimum inhibitory concentration (MIC) of the essential oil was determined, which corresponded to the lowest concentration at which no visible growth was detected in the Minimum Fungicidal Concentration (MFC).

### 3.8 Anticancer activity

Evaluating the anticancer characteristics of novel natural treatment methods on cell lines is a critical facet of cancer research. Various techniques, such as the MTT test, are often used to assess cell viability. Within the realm of breast cancer research, the cell lines MCF-7 and MDA-MB-231 have significant importance owing to their unique and distinguishing features. The citation is from Sommers et al. (1994). The MCF-7 cell line serves as a representative model for hormone-dependent breast cancer, distinguished by the presence of estrogen and progesterone receptors. These cells exhibit sensitivity to female sex hormones, namely, estrogen. Their level of aggression is often lower compared to the MDA-MB-231 line. The MCF-7 cell line is being used to investigate the processes involved in the progression of hormone-dependent malignancies and to assess the efficacy of therapies that specifically target these receptors. Conversely, the MDA-MB-231 cell line is a model for triple-negative breast cancer, which is distinguished by the lack of estrogen, progesterone, and HER2 receptors. This kind of cancer often exhibits heightened aggressiveness and poses challenges in terms of treatment. MDA-MB-231 cells are used as a model to investigate this specific form of breast cancer and assess novel treatment strategies that are not influenced by these receptors. The reference is from Bartucci et al. (2001).

The use of these 2 cell lines makes it possible to test the efficacy of different therapeutic approaches in models reflecting various clinical features of breast cancer, thus contributing to the advancement of research and development of treatments for this disease.

#### 3.8.1 Cell lines

The MCF-7 cell line, which expresses estrogen receptors (ER+), and the MDA-MB-231 cell line, which lacks estrogen receptors (ER-), were obtained from the American Tissue Culture Collection (ATCC, Molsheim, France), respectively, to mimic triple-negative breast cancer. A 5% CO2 environment at 37°C with humidity was used for the regulated cultivation of these cell lines. According to Jlizi et al. (2021), fetal calf serum (v/v) supplemented with 10% from Dutscher in Brumath, France, was added to RPMI 1640 culture medium.

#### 3.8.2 Cell viability determination

To determine cell viability, the cells being studied (1 × 104 cells per well) were exposed to different doses of CAEO (range from 3.12 to 200 μg/mL) on a 96-well culture plate for 24, 48, and 72 h. Before being exposed, CAEO was dissolved in dimethyl sulfoxide (DMSO). Following exposure, the cells were subjected to treatment with MTT reagent (0.5 mg/mL) at a temperature of 37°C for a duration of 4 h. Subsequently, the cells were washed with phosphate-buffered saline (PBS) and treated with DMSO. Absorbance measurements were taken at a wavelength of 570 nm (Yang et al., 2014). Each experiment was conducted in triplicate, with three wells per experiment. The GraphPad Prism version 8 software was employed to determine the 50% inhibitory concentration (IC50). Doxorubicin, a chemotherapeutic agent, was used as a positive control in the study, with each experiment also conducted in triplicate for IC50 determination using GraphPad Prism version 8 software.

## 4 Conclusion

In conclusion, research on the aerial component of Cistus albidus L., also known as the grey-leaved rockrose, has revealed substantial data concerning the potential health benefits and therapeutic qualities of its essential oil, CAEO. The composition of CAEO was completely characterised using Gas Chromatography-Mass Spectrometry (GC-MS), and camphene was identified as the main component. CAEO has demonstrated remarkable antioxidant potency, as seen by its low IC_50_ values in assays such as DPPH and β-carotene, indicating its ability to efficiently counteract oxidative stress. The results from the ABTS and total antioxidant capacity tests further highlight the robust antioxidant characteristics of CAEO, showcasing its importance as a natural source of antioxidants. In addition, the study has revealed the anti-inflammatory properties of the essential oil, specifically its capacity to hinder lipoxygenase, as well as its wide-ranging antimicrobial activities against different bacterial and fungal strains. Significantly, CAEO has exhibited a dose-dependent ability to suppress tumour cell growth, indicating potential uses in the realm of cancer investigation.

Furthermore, computational research have shed light on the physicochemical features, drug-like qualities, and pharmacokinetics of the various chemicals discovered in CAEO. Toxicity testing has supplied vital information about these chemicals’ safety. To summarize, our study shows that CAEO is a natural and safe reservoir of bioactive chemicals for both preventative and therapeutic uses, with no risk for toxicity.

## Data Availability

The original contributions presented in the study are included in the article/Supplementary Material, further inquiries can be directed to the corresponding authors.
