# Supplementary material for: Chemical profiling of volatile compounds of the essential oil of grey-leaved rockrose (Cistus albidus L.) and its antioxidant, anti-inflammatory, antibacterial, antifungal, and anticancer activity in vitro and in silico
Source: Front Chem. 2024 Feb 15;12:1334028. doi: 10.3389/fchem.2024.1334028 (PMC10905769; doi:10.3389/fchem.2024.1334028)
Supplement: Supplementary file 1 [file DataSheet1.docx]

*Supplementary file*

Chemical Profiling of Volatile Compounds of the Essential Oil of Grey-Leaved Rockrose (*Cistus albidus* L.) and Its Antioxidant, Anti-inflammatory, Antibacterial, Antifungal, and Anticancer Activity *In Vitro* and *In Silico*

**Table S1.** Molecular modeling targets and grid box characteristics.

| Proteins/PDB IDs | Grid Box Size | Gride Box Center | Native Ligand | Reference |
| --- | --- | --- | --- | --- |
| Glutathione Reductase / 3GRS | size_x = 40  size_y = 40  size_z = 40 | center_x = 70.597  center_y = 51.314  center_z = 18.685 | Butylhydroxytoluene (BHT) | [72] |
| Dihydrofolate reductase / 4M6J | size_x = 40  size_y = 40  size_z = 40 | center_x = 7.545  center_y = 7.421  center_z = -19.225 | Ciproflaxacin | [73] |
| Cytochrome P450 alpha-sterol demethylase / 1EA1 | size_x = 40  size_y = 40  size_z = 40 | center_x = 17.702  center_y = −3.978  center_z = 67.221 | Fluconazole | [61] |
| 5-Lipoxygenase / 1N8Q | size_x = 40  size_y = 40  size_z = 40 | center_x = 22.455  center_y = 1.2930  center_z = 20.362 | Protocatechuic Acid | [74] |
| Epidermal growth factor receptor (EGFR) / 1XKK | size_x = 40  size_y = 40  size_z = 40 | center_x = 19.273  center_y = 42.544  center_z = 36.837 | Vincristine | [75] |

Table S2. *In silico* drug-likeness, and the bioavailability of the identified components in CAEO. (1) Tricyclene, (2) α-Pinene, (3) Camphene, (4) β-Pinene, (5) *m*-Cymene, (6) Melonal, (7) Linalyl alcohol, (8) Camphor, (9) Borneol, (10) *cis-* β-Terpineol, (11) Linalool acetate, (12) Bornyl acetate.

| Prediction | 1 | 2 | 3 | 4 | 5 | 6 | 7 | 8 | 9 | 10 | 11 | 12 |  |
| --- | --- | --- | --- | --- | --- | --- | --- | --- | --- | --- | --- | --- | --- |
|  | Drug-Likeness Prediction | | | | | | | | | | | | |
| Lipinski | Yes | Yes | Yes | Yes | Yes | Yes | Yes | Yes | Yes | Yes | Yes | Yes |  |
| Egan | Yes | Yes | Yes | Yes | Yes | Yes | Yes | Yes | Yes | Yes | Yes | Yes |  |
| Veber | Yes | Yes | Yes | Yes | Yes | Yes | Yes | Yes | Yes | Yes | Yes | Yes |  |
| Bioavailability score | 0.55 | 0.55 | 0.55 | 0.55 | 0.55 | 0.55 | 0.55 | 0.55 | 0.55 | 0.55 | 0.55 | 0.55 |  |

Table S3. Evaluation of the toxicological properties of the major compounds in CAEO. (1) Tricyclene, (2) α-Pinene, (3) Camphene, (4) β-Pinene, (5) *m*-Cymene, (6) Melonal, (7) Linalyl alcohol, (8) Camphor, (9) Borneol, (10) *cis-* β-Terpineol, (11) Linalool acetate, (12) Bornyl acetate.

|  | **Hepatotoxicity** | | **Carcinogenicity** | | **Cytotoxicity** | | **Immunotoxicity** | | **Mutagenicity** | | **Predicted LD_50_ (mg/kg)** | **Class** |
| --- | --- | --- | --- | --- | --- | --- | --- | --- | --- | --- | --- | --- |
|  | **Pr** | **Pb** | **Pr** | **Pb** | **Pr** | **Pb** | **Pr** | **Pb** | **Pr** | **Pb** |  |  |
| **1** | Inact. | 0.90 | Inact. | 0.58 | Inact. | 0.98 | Inact. | 0.81 | Inact. | 0.77 | 15380 | VI |
| **2** | Inact. | 0.86 | Inact. | 0.60 | Inact. | 0.99 | Inact. | 0.93 | Inact. | 0.75 | 3700 | V |
| **3** | Inact. | 0.79 | Inact. | 0.56 | Inact. | 0.95 | Inact. | 0.91 | Inact. | 0.76 | 5000 | V |
| **4** | Inact. | 0.80 | Inact. | 0.66 | Inact. | 0.97 | Inact. | 0.95 | Inact. | 0.71 | 4700 | V |
| **5** | Inact. | 0.87 | **Act.** | **0.67** | Inact. | 0.98 | Inact. | 0.98 | Inact. | 0.89 | 2374 | V |
| **6** | Inact. | 0.69 | Inact. | 0.88 | Inact. | 0.82 | Inact. | 0.99 | Inact. | 0.98 | 5000 | V |
| **7** | Inact. | 0.76 | Inact. | 0.64 | Inact. | 0.82 | Inact. | 0.99 | Inact. | 0.95 | 2200 | V |
| **8** | Inact. | 0.72 | Inact. | 0.68 | Inact. | 0.61 | Inact. | 0.96 | Inact. | 0.94 | 775 | IV |
| **9** | Inact. | 0.77 | Inact. | 0.78 | Inact. | 0.88 | Inact. | 0.99 | Inact. | 0.98 | 500 | IV |
| **10** | Inact. | 0.75 | Inact. | 0.75 | Inact. | 0.89 | Inact. | 0.98 | Inact. | 0.90 | 2000 | IV |
| **11** | Inact. | 0.54 | Inact. | 0.62 | Inact. | 0.79 | Inact. | 0.99 | Inact. | 0.96 | 12000 | VI |
| **12** | Inact. | 0.58 | Inact. | 0.62 | Inact. | 0.67 | Inact. | 0.94 | Inact. | 0.94 | 3100 | V |

* Pr: Prediction, Pb: Probability, Inact.: Inactive, Act.: Active, Toxicity class explanation: IV: for substances that are harmful if swallowed (LD_50_ ranging from 300, and 2000 mg/kg), Class V: for compounds that may be harmful if swallowed (LD_50_ ranging between 2000, and 5000 mg/kg), Class VI: not harmful if swallowed (LD50 > 5000 mg/kg).
